# ABCC5 supports osteoclast formation and promotes breast cancer metastasis to bone

**DOI:** 10.1186/bcr3361

**Published:** 2012-11-22

**Authors:** Anna A Mourskaia, Eitan Amir, Zhifeng Dong, Kerstin Tiedemann, Sean Cory, Atilla Omeroglu, Nicholas Bertos, Véronique Ouellet, Mark Clemons, George L Scheffer, Morag Park, Michael Hallett, Svetlana V Komarova, Peter M Siegel

**Affiliations:** 1 Department of Medicine, McGill University, 1110 Pine Avenue West, Montreal, Quebec, H3A 1A3, Canada; 2 Faculty of Dentistry, McGill University, 3640 University Street, Montreal, Quebec, H3A 0C7, Canada; 3 Shriners Hospital for Children, 1529 Cedar Avenue, Montreal, Quebec, H3G 1A6, Canada; 4 Centre for Bioinformatics, McGill University, 3649 Promenade Sir William Osler, Montreal, Quebec, H3G 0B1, Canada; 5 Department of Pathology, McGill University, 3775 University Street, Montreal, Quebec, H3A 2B4, Canada; 6 Breast Cancer Functional Genomics Group, McGill University, 1160 Pine Avenue West, Montreal, Quebec, H3A 1A3, Canada; 7 Department of Biochemistry, McGill University, 3655 Promenade Sir William Osler, Montreal, Quebec, H3G 1Y6, Canada; 8 Department of Anatomy and Cell Biology, McGill University, 3640 University Street, Montreal, Quebec, H3A 2B2, Canada; 9 Goodman Cancer Research Centre, McGill University, 1160 Pine Avenue West, Montreal, Quebec, H3A 1A3, Canada; 10 Division of Medical Oncology, Princess Margaret Hospital, 610 University Avenue, Toronto, Ontario, M5T 2M9, Canada; 11 Division of Medical Oncology, Ottawa Hospital Cancer Centre, 501 Smyth Road, Ottawa, Ontario, K1H 8L6, Canada; 12 VU Medical Center, Department of Pathology, Postbus 7057 1007 MB, Amsterdam, The Netherlands

## Abstract

**Introduction:**

Bone is the most common site of breast cancer metastasis, and complications associated with bone metastases can lead to a significantly decreased patient quality of life. Thus, it is essential to gain a better understanding of the molecular mechanisms that underlie the emergence and growth of breast cancer skeletal metastases.

**Methods:**

To search for novel molecular mediators that influence breast cancer bone metastasis, we generated gene-expression profiles from laser-capture microdissected trephine biopsies of both breast cancer bone metastases and independent primary breast tumors that metastasized to bone. Bioinformatics analysis identified genes that are differentially expressed in breast cancer bone metastases compared with primary, bone-metastatic breast tumors.

**Results:**

ABCC5, an ATP-dependent transporter, was found to be overexpressed in breast cancer osseous metastases relative to primary breast tumors. In addition, ABCC5 was significantly upregulated in human and mouse breast cancer cell lines with high bone-metastatic potential. Stable knockdown of *ABCC5 *substantially reduced bone metastatic burden and osteolytic bone destruction in mice. The decrease in osteolysis was further associated with diminished osteoclast numbers *in vivo*. Finally, conditioned media from breast cancer cells with reduced ABCC5 expression failed to induce *in vitro *osteoclastogenesis to the same extent as conditioned media from breast cancer cells expressing ABCC5.

**Conclusions:**

Our data suggest that ABCC5 functions as a mediator of breast cancer skeletal metastasis. ABCC5 expression in breast cancer cells is important for efficient osteoclast-mediated bone resorption. Hence, ABCC5 may be a potential therapeutic target for breast cancer bone metastasis.

## Introduction

The skeleton is a favored site for breast cancer metastases due to unique features of the bone microenvironment, including the presence of growth factors and cytokines stored within the bone matrix [1]. The emergence of bone metastases disrupts normal bone homeostasis by perturbing interactions between bone-forming osteoblasts and bone-resorbing osteoclasts [2]. Breast cancer metastases in bone have typically been described as osteolytic in nature, and are associated with excessive bone destruction [3]. This ultimate shift toward bone resorption results from the ability of tumor cells, either directly or indirectly, to influence osteoclast differentiation and activity positively [4,5]. Subsequently, elevated bone resorption releases latent growth factors and cytokines that are stored in the bone matrix; these support tumor cell survival and growth that ultimately lead to further bone destruction [6,7]. Hence, the crosstalk between breast cancer cells and the bone microenvironment results in a vicious cycle of bone destruction and increased tumor growth in bone.

Breast tumors are heterogeneous, and cancer cells with bone-specific metastatic capabilities may preexist in the primary tumor. Indeed, gene signatures have been generated that predict whether a primary breast tumor will relapse to bone or visceral sites of metastasis [8]. A Src-related signature has also been proposed to segregate primary breast tumors based on their propensity to relapse to bone [9]. Numerous studies have identified cancer intrinsic factors that allow tumor cells to colonize and thrive in the bone microenvironment. *In vivo *selected breast cancer populations, isolated from bone metastases, have been used to identify unique functional mediators of bone metastasis [10-13].

These approaches have yielded valuable information regarding mechanisms involved in the spread, colonization, and growth of breast cancer cells in bone. However, growing evidence reveals discordance between the expression of specific markers in the primary breast tumor and those in the corresponding bone metastases [14]. Up to 40% of breast cancer patients displayed discordance in hormone-receptor expression between the primary tumor and the associated bone metastases [15,16]. Thus, it is likely that the bone microenvironment plays a considerable role in modulating the gene-expression profiles of breast cancer cells in emerging bone metastases. Hence, a number of important mediators of breast cancer skeletal metastasis will undoubtedly be overlooked in the analysis of primary breast tumors or breast cancer cells explanted *ex vivo *from bone metastases.

To circumvent these limitations, we sought novel mediators of skeletal metastasis directly in bone metastatic lesions from breast cancer patients. We applied laser-capture microdissection to isolate RNA from both trephine-biopsies of bone metastases and primary breast tumors. Numerous genes were differentially expressed between primary breast tumors that later relapsed to bone and breast cancer bone metastases, including several members of the ATP-binding cassette (ABC) transporter family that were overexpressed in the bone metastases relative to primary tumors. ABCC5 was found to be functionally involved in the formation of breast cancer bone metastases in two independent cell-based models.

## Materials and methods

### Primary breast tumor and bone metastases

Unguided or computed tomography (CT)-guided trephine biopsies were performed on breast cancer patients with known bone involvement at the Princess Margaret Hospital (Toronto, ONT, Canada), as previously described [17]. Biopsy material was immediately flash frozen and embedded in OCT compound. All procedures were performed with approval from the Research Ethics Board at the Princess Margaret Hospital. Primary breast tumor material was collected from patients who underwent surgery at the Montreal General or Royal Victoria Hospital (Montreal, QUE, Canada). Tumor banking was performed with approval from the Research Ethics Board of the McGill University Health Centre under the protocols SDR-99-780 and SDR-00-966. All patients provided written and informed consent.

### Laser-capture microdissection

Histologic sections of primary breast tumors or bone metastases were stained with H&E and examined by a clinical pathologist to identify regions within each section suitable for laser-capture microdissection (LCM). Sections (10 μm) were stained and dehydrated by using a HistoGene LCM Frozen Section Staining Kit (Cat KIT0401; Applied Biosystems, Carlsbad, CA, USA). Clusters of invasive mammary epithelial cells were identified and selected by using an ArcturusXT Microdissection System powered by ArcturusXT software v.1.1 (Applied Biosystems, Carlsbad, CA, USA). Breast cancer cells were captured by using an infrared laser adjusted to a diameter of 20 μm, laser power set to 65 mW and a duration of 20 msec, and pulsed through CapSure HS LCM Caps (Cat. LCM0214; Applied Biosystems, Carlsbad, CA, USA). The beam was passed over the sample to be collected with an overlap of 30% for each specimen. RNA was extracted from the microdissected cells by using a PicoPure RNA Extraction Kit (Cat. KIT0204; Applied Biosystems, Carlsbad, CA, USA) according to the manufacturer's instructions. RNA integrity and quantity was evaluated by using a 2100 Bioanalyzer platform (Agilent Technologies, Santa Clara, CA, USA).

### RNA amplification, labeling, and hybridization to Agilent microarray chips

Total RNA (1 to 2.5 ng) from microdissected material was subjected to two rounds of linear amplification by using a RiboAmp HS^Plus ^Amplification Kit (Cat. KIT0525; Applied Biosystems, Carlsbad, CA, USA), following the manufacturer's protocol. Profiles of resulting amplified RNA (aRNA) were assessed by using a 2100 Bioanalyzer (Agilent Technologies, Santa Clara, CA, USA). The aRNA samples (5 μg) were conjugated to Cy3 dye by using an Arcturus Turbo Labeling Kit (Cat. KIT0609; Applied Biosystems, Carlsbad, CA, USA). Universal human reference RNA (Stratagene) was amplified by using the same procedure and labeled with Cy5 dye (Cat, KIT0619; Applied Biosystems, Carlsbad, CA, USA). RNA concentration and dye incorporation was measured by using a Nanodrop ND-1000 UV-VIS spectrophotometer. Labeled RNA (0.825 μg) was then hybridized to 44K whole human genome microarray gene-expression chips (Cat. G4112F; Agilent Technologies, Santa Clara, CA, USA) by using a Gene Expression Hybridization Kit (Cat. 5188-5242; Agilent Technologies, Santa Clara, CA, USA) at 65°C for 17 hours according to the manufacturer's instructions. Microarray chips were washed, dried, and immediately scanned on a Microarray Scanner Model G2505B (Agilent Technologies, Santa Clara, CA, USA) by using Agilent Scanner Control Software vA7.0.1 (Agilent Technologies, Santa Clara, CA, USA).

### Gene-expression analysis

Microarray data were extracted by using Feature Extraction Software v. 9.5.3.1 (Agilent Technologies, Santa Clara, CA, USA). The raw data were then normalized, and differential expression was performed by using the LIMMA package in R/bioconductor [18]. Specifically, the arrays were normalized by using normexp background correction, loess within array, and quantile between array normalization. The *P *values for differential expression were adjusted for multiple testing. Candidate gene lists were generated by filtering the data on the basis of more than twofold difference in expression between bone metastases and primary breast tumors. The microarray data can be accessed through the GEO repository (ID GSE39494) [19].

### Real-time quantitative reverse-transcription polymerase chain reaction

RNA obtained after two rounds of amplification was quantified by using Quant-iT RiboGreen RNA Reagent based on the manufacturer's protocol (Cat. R11491; Invitrogen, Grand Island, NY, USA). Total RNA (25 ng) was converted to cDNA by using a Transcriptor Reverse Transcriptase kit (Cat. 048970300001; Roche, Laval, QUE, Canada) in accordance with the manufacturer's protocol. After reverse transcription, samples were subjected to real-time polymerase chain reaction (PCR) analysis by using SYBR Green PCR Master Mix (Cat. 04887352001; Roche, Laval, QUE, Canada). Primers were designed by using OligoPerfect software (Invitrogen, Burlington, ONT, Canada) in the region of the target gene surrounding the Agilent probes, at a concentration of 0.5 μ*M*. PCRs were performed on a LightCycler 480 system (Roche, Laval, QUE, Canada) under the following conditions: preincubation step (95°C for 10 minutes), 45-cycle amplification sequence (95°C for 10 seconds, 53°C for 10 seconds, 95°C for 6 seconds) and a melting step (95°C for 5 seconds, 65°C for 1 minute). A complete list of primer sequences can be found in the Supplementary Information. Results were analyzed with the absolute quantification method by using the second derivative maximum method feature of LightCycler 480 Software v. 1.5.0 SP4 (Roche, Laval, QUE, Canada).

### Immunohistochemistry

OCT-embedded primary breast tumors and bone trephine biopsies were sectioned (10 μm) and fixed in 2% paraformaldehyde. The sections were blocked with 2% BSA and 5% normal goat serum (NGS) and subsequently incubated overnight at 4°C with a primary antibody directed against ABCC5 (1:25; clone M5I-10). This monoclonal antibody was generated in Dr. Scheffer's laboratory (Amsterdam, The Netherlands) after injection of a bacterial fusion protein containing the N-terminal region of mouse ABCC5. The antibody recognizes mouse ABCC5 but also reacts strongly with the human orthologue. The sections were then incubated with Biotin-conjugated secondary antibody and developed with 3-3-diaminobenzidine-tetrahydrochloride (DAB). A standard hematoxylin counterstain was performed to demarcate cellular nuclei.

Primary mammary tumors and hindlimbs were excised from mice and fixed overnight in 4% paraformaldehyde. Bones were decalcified in a solution of 14.5% ethylenediaminetetraacetic acid (EDTA) and 15% glycerol for 4 weeks. Tissues were then paraffin embedded and sectioned. Sections (5 μm) were deparaffinized and stained with a freshly prepared tartrate-resistant acid phosphatase (TRAP) staining solution (naphthol AS-TR Phosphate, fast blue RR salt, and sodium tartrate). Slides were scanned by using a Scanscope XT digital slide scanner (Aperio, Vista, CA, USA) and analyzed with Imagescope software (Aperio, Vista, CA, USA). The number of TRAP-positive cells within breast cancer lesions in bone was counted manually and is presented as the number of osteoclasts per square millimeter of tumor mass.

### Immunoblotting

Human and mouse cell lines were lysed in TNE lysis buffer, as previously described [20]. Total protein concentrations were determined with the Bradford Protein Assay (Cat. 500-006; Bio-Rad Laboratories, Mississauga, ONT, Canada) and 20 to 50 μg of protein was separated with sodium dodecylsulfate polyacrylamide gel electrophoresis (SDS-PAGE) and transferred to polyvinylidene fluoride (PVDF) membranes (Cat. IPVH00010; Millipore, Billerica, MA, USA). The membranes were blocked in 5% wt/vol nonfat dry milk containing 0.1% Tween and incubated with the following primary antibodies: ABCC5 (1:100 dilution; M5I-10) and α-tubulin (1:20,000 dilution; Cat. T9026; Sigma, Oakville, ONT, Canada). The blots were then incubated with horseradish-peroxidase-conjugated secondary antibodies, and proteins were visualized with an enhanced chemiluminescence detection system (Cat. 34080; Pierce, Nepean, ONT, Canada).

### DNA constructs

Short-hairpin RNA (shRNA) sequences targeting the human and mouse *ABCC5 *mRNA, as well as the scrambled control sequence, were designed by using the RNAi central website at Cold Spring Harbor Laboratories [21]. The sequences of the shRNAs used for *ABCC5 *knockdown are as follows (target sequence denoted in **bold **text: h, human; m, mouse): *hABCC5 *sh: TGC TGT TGA CAG TGA GCG **ACC TCA AAG TCT GCA ACT TTA A**TA GTG AAG CCA CAG ATG TAT TAA AGT TGC AGA CTT TGA GGG TGC CTA CTG CCT GGA; *mabcc5 *sh1: TGC TGT TGA CAG TGA GCG **ACC TCA TCC TGT CCT GCT GAA A**TA GTG AAG CCA CAG ATG TAT TTC AGC AGG ACA GGA TGA GGG TGC CTA CTG CCT CGG A; *mabcc5 *sh2: TGC TGT TGA CAG TGA GCG **CCC TGA CTA TGG CAT TCA AGA A**TA GTG AAG CCA CAG ATG TAT TCT TGA ATG CCA TAG TCA GGA TGC CTA CTG CCT CGG A; scrambled sh: TGC TGT TGA CAG TGA GCG **AAG TCC ATA CTT AGT CGA TAG A**TA GTG AAG CCA CAG ATG TAT CTA TCG ACT AAG TAT GGA CTC TGC CTA CTG CCT CGG A. These sequences were PCR amplified, digested, and cloned into the LMP vector as *Xho*I/*Eco*RI fragments by following published instructions [22].

### Cell culture and *in vitro *osteoclastogenesis assay

Parental MDA-MB-231 breast cancer cells were obtained from the American Type Culture Collection and transduced with a triple reporter system, as previously described [23]. The MDA-MB-231-derived bone metastatic (1833-BM1) and lung metastatic (4175-LM2) populations were derived as described previously [10,24]. Human breast cancer cell lines were cultured in DMEM supplemented with 10% fetal bovine serum and MEM Nonessential Amino Acids (1X), gentamycin, and amphotericin B. The 4T1 murine mammary carcinoma cell line was obtained from the American Type Culture Collection. Nonmetastatic 67NR and lung-metastatic 66cl4 murine mammary carcinoma cell lines [25] were kindly provided by Dr. Fred Miller (Barbara Ann Karmanos Cancer Institute, Detroit, MI, USA) and cultured in DMEM supplemented with 10% fetal bovine serum, 10 m*M *HEPES, 1 m*M *sodium pyruvate, 1.5 g/L sodium bicarbonate, gentamycin, and amphotericin B.

For the *in vitro *osteoclastogenesis assay, 5 × 10^5 ^4T1-derivative cells were plated in 10-cm cell-culture dishes. The following day, media were changed to DMEM supplemented with 10% fetal bovine serum and subsequently conditioned for 48 hours. Protocols used to establish primary osteoclast cultures from BALB/c mice were performed in accordance with the McGill University guidelines established by the Canadian Council on Animal Care. Bone marrow was collected from tibiae and femurs of mice (BALB/c, male, 6 weeks old; Charles River, Wilmington, MA, USA), as described previously [26], and cultured in 75-cm^2 ^tissue culture flasks (15 × 10^6 ^cells per flask) in α-minimal essential medium supplemented with 10% fetal calf serum, penicillin, streptomycin, and 25 ng/ml of human recombinant macrophage-colony stimulating (M-CSF) factor (Cat. 300-25; PeproTech, Inc.). On day 1, nonadherent cells were collected, plated at 5 × 10^3 ^cells/cm^2^, and supplemented with M-CSF (50 ng/ml) and recombinant GST-RANKL (100 ng/ml). On day 4, fresh media with or without RANKL (100 ng/ml) or conditioned media harvested from 4T1-derivatives (10%) were added to the cultures. M-CSF (50 ng/ml) was present in all conditions tested. On day 6, cells were fixed with 4% paraformaldehyde (10 minutes), washed with phosphate-buffered saline, and stained for tartrate-resistant acid phosphatase (TRAP) (Cat. 387A-KT; Sigma, Oakville, ONT, Canada).

### Left cardiac ventricle injections

Female SCID/beige and BALB/c mice (4 to 6 weeks old) were purchased from Charles River Laboratories. The animals were housed in facilities managed by the McGill University Animal Resources Centre. All animal experiments were conducted under a McGill University-approved Animal Use Protocol in accordance with guidelines established by the Canadian Council on Animal Care. Breast cancer cells were harvested from subconfluent cultures and resuspended in sterile PBS. Mice were anesthetized with isofluorane, and 1 × 10^5 ^human or mouse breast cancer cells, in a volume of 100 μl, were injected into the left cardiac ventricle by using 26G needles [12]. A successful injection was distinguished by the pumping of arterial blood into the syringe during the injection procedure and confirmed by a uniform luminescent signal throughout the entire animal body after 1833-BM1 cell inoculation.

### *In vivo *bioluminescent imaging

Tumor outgrowth within skeletal sites of mice injected with 1833-BM1 breast cancer cells was monitored by using an IVIS 100 (Caliper Life Sciences, Hopkinton, MA, USA) bioluminescence imaging system, as previously described [27]. The resulting data were normalized to the signal generated by the initial cell inoculum, which was measured immediately after cardiac injection. Total metastatic burden was measured by setting a uniform scale for each group of mice, outlining regions of interest around all luminescence signals in the body and summing them.

### X-ray microcomputed tomography (μCT) imaging

At the end of the cardiac-injection experiments (21 days for 1833-BM1 and 13 days for the 4T1 models), mice were anesthetized and immobilized with tape in the imaging tube of a Skyscan 1178 μCT. All images were obtained with an x-ray source operating at 50 kV (1833-BM1) or 45 kV (4T1) and 615 mA, with an exposure time of 480 msec. Animals were rotated through 180 degrees at a rotation step of 1.26 degrees (1833-BM1 cells) and 0.9 degrees (4T1 cells). Cross-section images from tomography projection images were reconstructed by using the NRecon program package v.1.6.4.7 (SkyScan, Kontich, Belgium). Reconstruction parameters, including smoothing (1), ring artefacts reduction (4), and beam-hardening correction (30%), were fixed for all the samples. The dynamic image range was defined between 0 and 0.045 for all the samples. Bone alignment was adjusted in all specimens by using DataViewer v.1.4.3.2 (SkyScan, Kontich, Belgium). Bone volume was determined in 3D by using CTAn software v.1.11.8.0 (SkyScan, Kontich, Belgium). In brief, for each bone, a volume of interest (VOI) was determined starting under the growth plate and extending 20 (femur) and 25 (tibia) sections below the diaphysis. For each model, the VOI was designed by drawing user-defined polygons on the 2D sections that encompass the bone of interest. In the binary image mode, the histogram was set at minimum 100 to maximum 255 for a given dataset for each specimen. Each 3D model was visualized by using CTvox v.2.3 (SkyScan, Kontich, Belgium). The absolute bone volume was determined for each piece of bone and expressed in cubic millimeters. Control groups, including uninjected mice of similar strain and age as the experimental animals, were used as reference for normal bone volume. The degree of bone destruction was determined as percentage difference between the average bone volumes in experimental groups compared with those in the appropriate control cohorts.

### Statistical analysis

Statistical significance values for RT-qPCR expression, whole body luminescence, μCT bone volumes, and osteoclast assays were obtained by performing a two-sample variance two-tailed Student *t *test.

Supplemental Materials and Methods can be found as Additional file 1.

## Results

### Laser-capture microdissection of human breast cancer bone metastases

To generate gene-expression profiles of primary human breast cancer bone metastases and primary breast cancers, we selected five fresh-frozen samples for each group (see Additional file 2, Additional file 3). For this study, the primary breast tumors were not patient-matched to the bone metastases.

The bone metastases were obtained from breast cancer patients who had consented to undergo unguided or CT-guided trephine biopsies. To ensure that the biopsy material contained breast cancer cells, we performed co-immunofluorescence staining with both luminal (CK8/18) and basal (CK5) cytokeratins. Clusters of CK5-positive epithelial cells were found in all of the bone metastases used in our analysis, a marker associated with basal-like breast cancer cells (see Additional file 4, top panels). However, in three of five samples (BM-001, BB-005, BM-007), expression of the luminal cytokeratin 8/18 markers were also detected, suggesting that these breast cancer cells were of mixed lineage (Additional file 4, middle panels). A DAPI stain identified the location of all cells in each section (Additional file 4, bottom panels).

**Figure 1:**
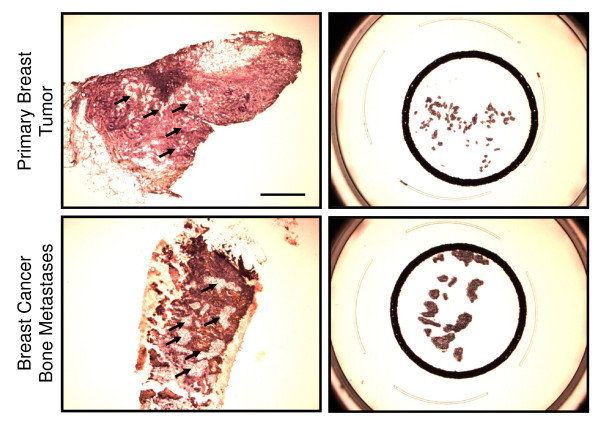
**Laser-capture microdissection (LCM) of breast cancer cells**. Malignant mammary epithelial cells were isolated from primary breast tumors (top panels) and bone trephines (bottom panels). The selected regions containing the breast cancer cells were adhered to LCM caps (right panels). Left panels represent images of slides after completion of LCM, with black arrows indicating the isolated material. The scale bar on the top left image represents 1,000 μm and applies to all panels.

Clusters of invasive epithelial cells were specifically identified in each sample and subjected to laser-capture microdissection (LCM) (Figure 1). After RNA extraction, two rounds of amplification and labeling, gene-expression profiles of each sample were obtained by using human whole genome (44K) Agilent microarray chips. Unsupervised clustering using the top 200 most-variable probes segregated the samples into primary breast tumors and bone metastases, irrespective of ER, PR, and Her2 status (data not shown).

### Identification of putative molecular mediators of breast cancer skeletal metastasis

Filtering the differential gene-expression data yielded a list of 244 overexpressed and 185 underexpressed probes in breast cancer skeletal metastases compared with primary breast tumors. After a review of the literature, 118 upregulated and 82 downregulated unique genes with known or ascribed functions were identified in bone metastases compared with primary tumors (see Additional file 5). These genes were further categorized into distinct functional groups based on their proposed roles, which included mediators of: transcription, translation, posttranslational modification, mitochondrial functions/metabolism, cell-cycle progression, cellular transport, cytoskeletal organization, cellular adhesion/migration/invasion, tumor microenvironment, chemotaxis, cell differentiation, apoptosis/survival, and other (see Additional file 6).

A number of genes responsible for cellular migration and invasiveness were downregulated in bone metastases compared with primary tumors (*NCK2 *[28], *PHPT1 *[29], *LIMK1 *[30], *TESK1 *[31], and *TMSB10 *[32]). Moreover, a few genes involved in modulation of the extracellular matrix (*ADAMTS4 *[33], *MMP1 *[34], *MMP3 *[35], and angiogenesis (*MFAP5 *[36], *SFRP2 *[37], *SRPK1 *[38], *VEGF *[39], and *CHI3L1 *[40]) were found to be expressed at higher levels in the primary breast tumors compared with the bone metastases. Our data support a role for these two gene sets in promoting local invasion at the primary tumor, which may have facilitated breast cancer dissemination to distant organs such as the bone.

Interestingly, the expression of genes (*GFRA1*, *ID1, ABCG2*) implicated in the regulation of hematopoietic progenitor cells was found to be elevated in bone metastases compared with the primary breast tumors that had relapsed to bone [41-44]. This finding lends support to the notion that breast cancer cells may use the endosteal niche, which preexists within the bone microenvironment, to colonize the bone [45]. It has been reported that cancer cells potentially compete with HSCs for occupancy of these endosteal niches and represent privileged sites where cancer cells first establish [46]. Additionally, a number of prosurvival (*BCL2 *[47], *PPEF2 *[48]) and proapoptosis (*BID *[49], *HEBP2 *[50], and *PKNOX1 *[51]) genes were up- and downregulated, respectively, in bone metastases compared with primary breast tumors. Importantly, the expression of well-known pro- (*BCL2*) and anti-apoptotic (*BID*) genes was validated with RT-qPCR (see Additional file 7, Additional file 8).

Finally, macrophage inhibitory factor (*MIF*) was found to be downregulated in breast cancer skeletal metastases compared with the primary mammary tumors. It has been shown that MIF promotes breast cancer cell proliferation [52,53], angiogenesis [54], and survival [55]. However, although MIF was frequently overexpressed in primary mammary tumor tissues, its presence was inversely correlated with the nodal spread of breast cancer [56].

A recent study suggested that intracellular MIF levels are low in aggressive breast cancer cell lines and that cytosolic MIF expression associates with an increase of recurrence-free survival patients, whereas extracellular MIF promotes migration and invasion of breast cancer cells [57]. Interestingly, MIF was also shown to inhibit osteoclastogenesis in the *in vitro *and *in vivo *assays [58]. Thus, loss of MIF may be required during the formation of osteolytic bone metastases.

By using RT-qPCR analysis, we were able to validate the gene-expression changes for several candidates initially identified with microarray-based gene-expression profiling (see Additional file 7, Additional file 8). Intriguingly, several members of the ATP-binding cassette (ABC) transporter superfamily (*ABCA5*, *ABCC5*, and *ABCG2*) were consistently overexpressed in breast cancer bone metastases compared with primary breast tumors (Additional file 7, Additional file 8). Although a significant body of literature has linked ABCG2 expression with drug resistance in breast cancer [59], considerably less is known about ABCA5 and ABCC5 in this disease.

### ABCC5 is overexpressed in breast cancer bone metastases and bone metastatic breast cancer cells

**Figure 2:**
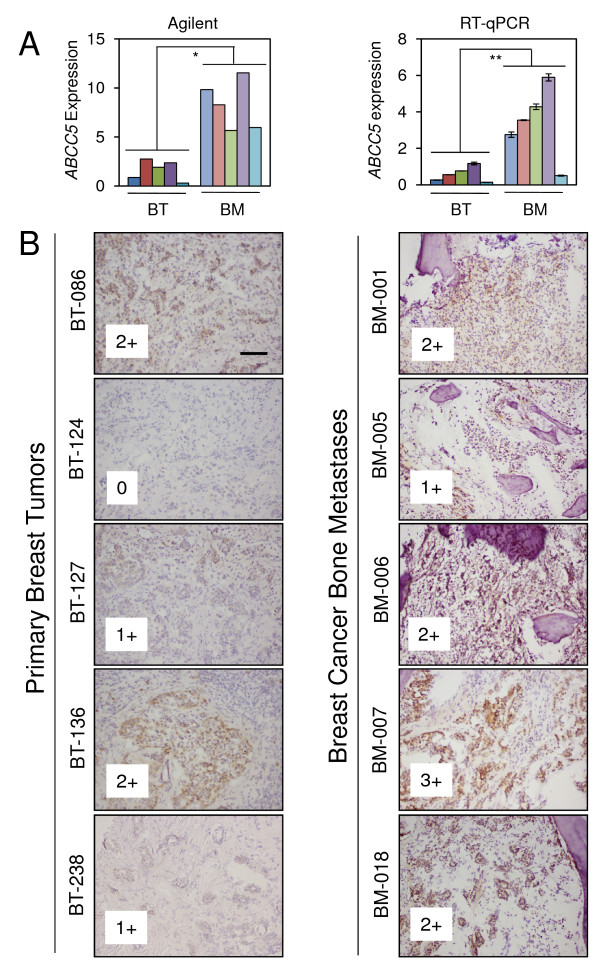
***ABCC5 *expression is elevated in breast cancer bone metastases**. **(A) ***ABCC5 *gene expression was found to be significantly overexpressed in breast cancer bone metastases (BMs) compared with primary breast tumors (BTs) that were metastatic to bone by both Agilent microarray analysis (left panel) and RT-qPCR (right panel); *P = 0.002; **P = 0.03. **(B) **Immunohistochemical (IHC) analysis confirmed that ABCC5 expression was higher in bone metastases (right panels) compared with primary breast tumors (left panels). IHC was performed on frozen sections for all the samples, and the ABCC5 staining intensity is indicated in the lower left corner of each image. Scale bar in the first panel represents 100 μm and applies to all images.

Given the availability of antibody reagents, we focused our attention on *ABCC5*, a candidate gene that was found to be significantly overexpressed in breast cancer skeletal metastases relative to primary tumors by both microarray-based and RT-qPCR methods (Figure 2A). Furthermore, immunohistochemical analysis revealed that ABCC5 protein levels appeared to be enriched in breast cancer bone metastases compared with primary mammary tumors (Figure 2B).

**Figure 3:**
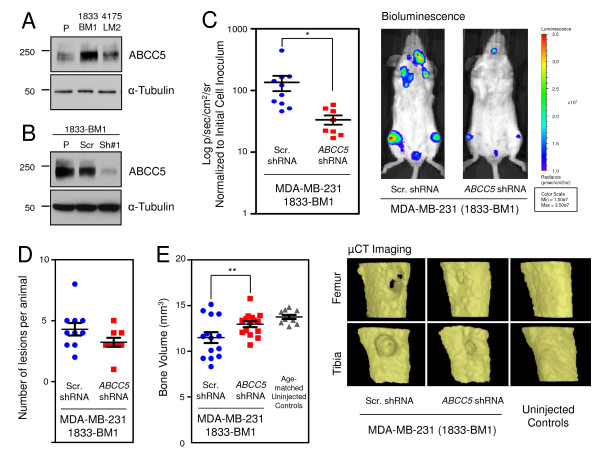
**ABCC5 promotes the formation of osteolytic bone metastases in MDA-MB-231 human breast cancer cells**. **(A) **ABCC5 expression is appreciably higher in the bone metastatic 1833-BM1 population compared with parental MDA-MB-231 cells and the lung-metastatic 4175-LM2 population. **(B) **Immunoblot analysis reveals that ABCC5 expression was efficiently silenced via short-hairpin RNA (shRNA) in the 1833-BM1 breast cancer cell line. An immunoblot for α-tubulin served as a loading control in panels (A) and (B). **(C) **Diminished ABCC5 expression in 1833-BM1 cells resulted in reduced formation of skeletal metastases after cardiac injection, as determined by *in vivo *bioluminescent imaging. The data and representative images are shown for day 21 after tumor-cell injection (scr shRNA, *n *= 10; *ABCC5 *shRNA, *n *= 8; *P = 0.024). **(D) **All of the animals developed osteolytic lesions after injection of the breast cancer cells. The number of lesions per animal was determined by analyzing blinded μCT images. **(E) **The degree of osteolytic bone destruction was quantified by *in vivo *μCT imaging. Bone volume was calculated from reconstructions of defined regions of both the proximal tibia and femur from mice imaged on day 21 after injection (scr shRNA, *n *= 20; *ABCC5 *shRNA, *n *= 16; **P = 0.041). Controls (*n *= 10) refers to age-matched females mice that were not injected with breast cancer cells. Representative images of bone reconstructions are shown.

**Figure 4:**
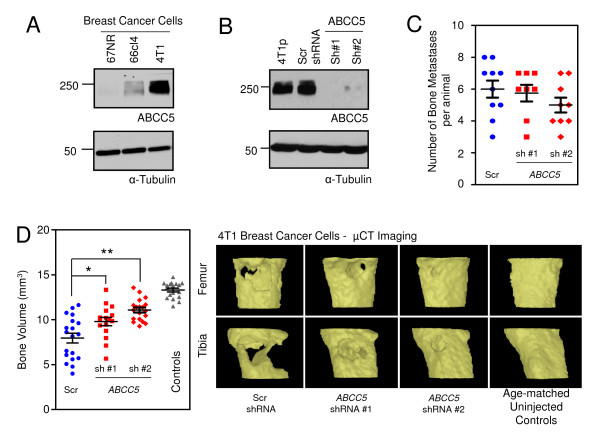
**ABCC5 promotes the formation of osteolytic bone metastases induced by 4T1 murine breast cancer cells**. **(A) **ABCC5 expression is considerably elevated in the highly metastatic mouse breast cancer cell line 4T1, which exhibits bone metastatic activity, when compared with a less-metastatic 66cl4 (lung) and nonmetastatic 67NR populations. **(B) **ABCC5 expression was stably silenced by using two independent short-hairpin RNAs (sh 1, sh 2) in 4T1 breast cancer cells, as demonstrated with immunoblot analysis, when compared with parental 4T1 cells (4T1p) and a scrambled shRNA control (Scr shRNA). **(C) **All of the animals had osteolytic lesions after breast cancer cell injections. The number of lesions per animal is indicated and was established by examining blinded μCT images. **(D) **Reduction in ABCC5 levels in 4T1 breast cancer cells results in the diminished formation of osteolytic bone metastases after cardiac injection (scr shRNA, *n *= 19; *abcc5 *shRNA 1, *n *= 16; *abcc5 *shRNA 2, *n *= 19; *P = 0.015; **P < 0.001). Controls (*n *= 18) refers to age-matched female mice that were not injected with breast cancer cells. The degree of osteolytic bone destruction in the hindlimbs was quantified by *in vivo *μCT imaging. Bone volume of defined regions of both the proximal tibia and femur is shown for animals imaged on day 21 after injection. Representative images of bone reconstructions are shown.

As a first step to validate functionally a role for ABCC5 in promoting bone metastasis, we interrogated the expression of ABCC5 in breast cancer cell lines with high bone-metastatic potential. By using a series of *in vivo*-selected MDA-MB-231 breast cancer cell populations that are metastatic to bone and lung, we demonstrated that ABCC5 expression was highest in bone metastatic cell populations (1833-BM1) when compared with lung metastatic cells (4175-LM2) or the parental MDA-MB-231 cell line (Figure 3A). We also examined an independent murine breast cancer series isolated from the same original mammary tumor that are either nonmetastatic (67NR), lung-metastatic (66cl4), or metastatic to multiple organs, including bone (4T1). We demonstrated that ABCC5 expression was considerably higher in the bone-metastatic 4T1 cell line relative to lung-metastatic 66cl4 and nonmetastatic 67NR populations (Figure 4A).

### Loss of ABCC5 expression diminishes the formation of breast cancer bone metastases

To determine whether ABCC5 is important for the ability of breast cancer cells to metastasize to bone, we stably diminished ABCC5 expression in bone-metastatic breast cancer cell lines by using short-hairpin RNAs (shRNAs). An efficient and stable knockdown was achieved in both human 1833-BM1 (Figure 3B) and mouse 4T1 (Figure 4B) breast cancer cell lines relative to the parental cell lines or populations harboring a scrambled shRNA control. Importantly, diminished ABCC5 expression in human and mouse mammary cancer cell lines did not affect their proliferative properties *in vitro *or their *in vivo *growth as mammary tumors (Additional file 9). However, loss of ABCC5 expression in the human 1833-BM1 population resulted in a fourfold reduction in overall bone metastatic burden after left cardiac ventricle injections, as measured by bioluminescence imaging (Figure 3C). Whereas the total number of bone metastases per mouse was similar in scrambled control and *ABCC5 *shRNA cohorts (Figure 3D), the size of the individual lesions was clearly diminished (Figure 3C). Interestingly, the osteolytic bone-metastatic lesions arising in mice injected with ABCC5^low ^1833-BM1 cells were significantly smaller (6% bone destruction) than were those observed in animals injected with the scrambled shRNA-expressing population (16% bone destruction), as measured with volumetric μCT analysis (Figure 3E).

To confirm this functional role for ABCC5 in promoting breast cancer metastasis to bone, we analyzed the effects of reduced ABCC5 expression on the ability of 4T1 breast cancer cells to form bone metastases after cardiac injection. Two independent 4T1 populations, each harboring a unique shRNA targeting *ABCC5*, and 4T1 cells containing a scrambled shRNA, were injected into separate cohorts of mice. Similar to our results with the human breast cancer cell-line model, suppression of ABCC5 expression in mouse breast cancer cell lines did not result in the alteration of the total number of bone metastases per mouse (Figure 4C). However, μCT analysis revealed that reduced *ABCC5 *expression in mouse-derived 4T1 cells resulted in significantly less (shRNA 1, 26% bone destruction; shRNA 2, 16% bone destruction) bone destruction compared with the scrambled controls (40% bone destruction) (Figure 4D). Interestingly, no difference was found in either the number of spontaneous lung metastases or the size of the lesions formed by 4T1-derivative breast cancer cells harboring the ABCC5 knockdown when compared with cells expressing the scrambled shRNA (see Additional file 10). Together, these results support an important functional role for ABCC5 in promoting the colonization and growth of breast cancer cells specifically within the bone.

### Proliferation and apoptosis are not affected in mammary tumors or bone metastases by diminished ABCC5 expression

To investigate potential explanations for the reduction in the size of osteolytic lesions arising in mice injected with breast cancer cells engineered to express ABCC5 shRNAs, we examined the proliferative and apoptotic indices in both mammary tumors and bone metastases. In agreement with the observation that reduced ABCC5 levels did not negatively influence primary tumor growth (Additional file 9), we did not observe statistically significant differences in the percentage of Ki67-positive nuclei in primary tumors derived from 1833-BM1 or 4T1 cells, possessing normal or shRNA-reduced levels of ABCC5 (see Additional file 11A and B). Interestingly, no differences in proliferation were observed in bone metastases that formed in mice injected with 1833-BM1- or 4T1-derived breast cancer populations, which possessed diminished ABCC5 expression or normal endogenous levels of ABCC5 (Additional file 11C and D).

To determine whether loss of ABCC5 was associated with elevated levels of apoptosis, we performed immunohistochemical staining for cleaved caspase-3 in both mammary tumors and bone metastases that arose in mice injected with 1833-BM1- or 4T1-derived breast cancer populations. Diminished ABCC5 expression did not result in elevated apoptosis in either end-stage mammary tumors (Additional file 12A and B) or end-stage osteolytic bone metastases (Additional file 12C and D).

### Reduced ABCC5 levels in breast cancer cells correlate with reduced osteoclast numbers within bone metastatic lesions and diminished *in vitro *osteoclastogenesis

**Figure 5:**
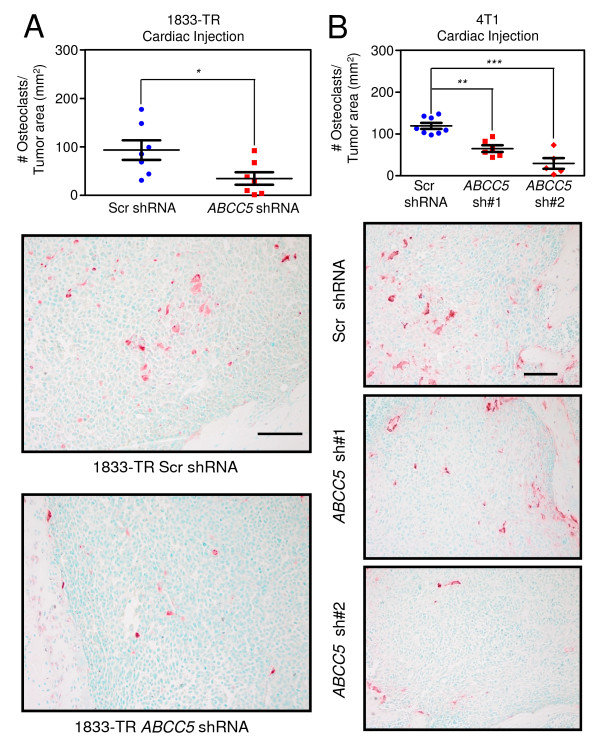
**Loss of ABCC5 in breast cancer cells results in diminished osteoclast numbers within osteolytic lesions**. **(A) **Quantification of TRAP-positive osteoclasts within osteolytic lesions formed by human-derived 1833-BM1 cells (scr shRNA, *n *= 7; *ABCC5 *shRNA, *n *= 7; *P = 0.03). Representative images are shown for each group. The scale bars represent 100 μm and apply to both images. **(B) **Quantification of TRAP-positive cells present within bone metastases formed in mice injected with 4T1-derived populations (scr shRNA, *n *= 8; *abcc5 *shRNA 1, *n *= 6; *abcc5 *shRNA 2, *n *= 5; **P < 0.001; ***P < 0.001). Representative images are shown for each group. The scale bar in the upper image represents 100 μm and applies to all images.

To better define the mechanisms responsible for impaired bone metastasis in mice injected with the *ABCC5*-knockdown breast cancer cell populations, we examined the density of osteoclasts present within lytic lesions formed by the MDA-MB-231 and 4T1-derived cells. Representative bone sections were stained for tartrate resistant acid phosphatase (TRAP), an osteoclast-specific marker. The number of TRAP-positive cells was significantly reduced by 2.7 times in the 1833-BM1 lesions expressing the *ABCC5 *shRNA compared with the bone metastases harboring the scrambled shRNA control (Figure 5A). Two independent 4T1-derived populations, each expressing a unique shRNA-targeting *abcc5*, also showed a 1.8- and 4.1-fold reduction in TRAP-positive cells when compared with 4T1 breast cancer cells harboring the scrambled shRNA control (Figure 5B). Together, these data demonstrate that loss of ABCC5 function in breast cancer cells results in decreased osteoclast numbers within bone-metastatic lesions. These results are in agreement with the diminished capacity to form osteolytic metastases that is exhibited by breast cancer cells with diminished ABCC5 expression. Our data support a role for ABCC5 in promoting the specific growth of breast cancer cells within the bone microenvironment through a mechanism that favors osteoclast formation and/or function.

**Figure 6:**
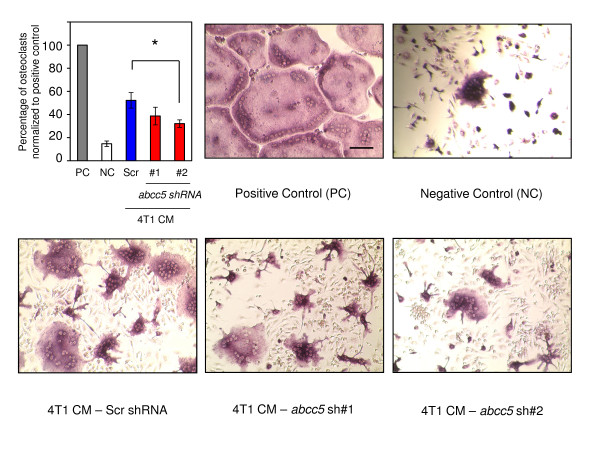
**Loss of ABCC5 in breast cancer cells results in diminished osteoclast differentiation *in vitro***. Mouse bone marrow cells were primed with M-CSF and RANKL for 3 days and then cultured for 2 days untreated (negative control, NC), RANKL treated (positive control, PC), or conditioned media from 4T1-derived populations (scrambled shRNA (Scr), abcc5 shRNA 1, and abcc5 shRNA 2). Osteoclasts were counted for each condition, and the results are expressed as a percentage of the osteoclast number in the positive control. The data are presented as averages of five independent experiments (*P < 0.05). Representative images are presented for each group. The scale bar in the first image represents 100 μm and applies to all images.

Further to investigate the potential role of tumor-intrinsic ABCC5 in promoting osteolytic lesion formation, we performed an *in vitro *osteoclast differentiation assay. Primary osteoclast cultures were established from murine bone marrow and primed with macrophage colony-stimulating factor (M-CSF) and receptor activator of nuclear factor κ-B ligand (RANKL). The osteoclast precursors were subsequently stimulated with conditioned media from the 4T1-derived populations, which exhibited the most-pronounced difference in osteoclast numbers between control and ABCC5 knockdown breast cancer cells *in vivo *(Figure 5). Intriguingly, the number of multinucleated TRAP-positive osteoclasts was reduced by 1.3 and 1.6 times when osteoclast precursors were cultured in media collected from 4T1 harboring two independent *abcc5*-specific shRNAs, when compared with the scrambled shRNA control (Figure 6).

## Discussion

The vicious cycle of bone-metastasis formation argues that breast cancer cells colonizing the bone are influenced by growth factors that are stored in the bone matrix; these are released during bone resorption [1,2]. These influences predict that breast cancer bone metastases will differ in their gene-expression profiles from primary breast tumors. To evaluate gene-expression profiles of *in situ *bone metastases from breast cancer patients, we performed laser-capture microdissection (LCM) on trephine biopsies and compared them with profiles obtained from LCM material of primary breast tumors. Numerous genes were differentially expressed between primary breast tumors with known relapse to bone and breast cancer bone metastases and were categorized according to their known or proposed functions.

Intriguingly, three members of the ATP-binding cassette (ABC) transporter family (*ABCA5, ABCC5, ABCG2*) were overexpressed in breast cancer bone metastases compared with primary tumors that are metastatic to bone (Additional file 7 and Additional file 8). The human genome encodes 49 ABC genes that are arranged in seven subfamilies designated A to G [60]. These genes encode ATP energy-dependent molecular pumps that transport substrates across membranes, either in or out of cells or into cellular vesicles, against their electrochemical gradient [61]. Consistent with the role of these transporters in the excretion of diverse compounds, their expression (ABCG2, ABCA1, ABCA7, ABCG1, and ABCG5) has been found in lactating mammary epithelium [62,63]. Furthermore, the ABC transporters (ABCG2, ABCB1) have been proposed to be expressed by quiescent cancer stem cells, which allows them to survive cytotoxic or targeted therapies leading to relapse [64].

We focused on ABCC5 (MRP5) as a candidate mediator of breast cancer skeletal metastases because we validated its expression in bone metastases at both the mRNA and protein levels (Figure 2). ABCC5 represents a membrane-spanning protein belonging to the C subfamily of the ABC transporters, with the ability to transport endogenous cyclic nucleotides [65]. One concern associated with our identification of ABC transporters was the possibility that these proteins were upregulated as a consequence of the patient's treatment history rather than any specific role that they might play in the establishment of bone metastases [66,67]. To address this possibility, we examined ABCC5 expression in breast cancer cell populations that exhibit a bone-metastatic phenotype. We first used a series of *in vivo *selected MDA-MB-231 cell lines with different organ-specific metastatic potential and found that ABCC5 was most highly expressed in the bone-metastatic MDA-MB-231 cells when compared with the parental and lung-tropic MDA-MB-231 cells (Figure 3A).

We next determined the level of ABCC5 expression in mouse-derived breast cancer cells with differential metastatic abilities. Consistent with the result in MDA-MB-231 cells, the highly metastatic 4T1 population, which is highly metastatic to bone and other sites, exhibited considerably higher ABCC5 expression in comparison with lung-only metastatic 66cl4 and nonmetastatic 67NR populations (Figure 4A). These data reveal that ABCC5 expression is highest in breast cancer cells that display enhanced bone-metastatic phenotypes, under conditions in which no treatments (antiestrogens, chemotherapy, or bisphosphonates) were used. Thus, it is conceivable that elevated ABCC5 expression is selected for because of a particular function that enables breast cancer cells to colonize the bone microenvironment efficiently.

The physiological functions of ABCC5 are, at present, poorly defined. It was reported that this protein serves as an efflux pump for intracellular cyclic guanosine monophosphate (cGMP) and, to a lesser degree, cyclic adenosine monophosphate (cAMP) [65]. A growing body of literature suggests that elevated intracellular cGMP levels result in reduced breast cancer cell proliferation, ultimately triggering apoptosis via activation of protein kinase G (PKG) [68-70]. Nitric oxide and natriuretic peptides, which serve as activators of soluble and transmembrane guanylyl cyclises, respectively, are abundant in bone and exert complex effects on bone cells, bone turnover, and bone formation [71]. Thus, we reasoned that upregulation of a cGMP efflux pump could be advantageous for breast cancer growth and survival in the bone microenvironment. Diminished ABCC5 expression resulted in a significant reduction in the size of osteolytic lesions formed by both 1833-BM1 and 4T1 breast cancer cell models when compared with controls. However, this reduction was not a reflection of general alterations in breast tumor cell growth (Additional file 9). Consistently, no change in breast cancer cell proliferation, in either primary breast tumors or bone metastases, was noted when ABCC5 was knocked down compared with controls (Additional file 11). Moreover, the removal of ABCC5 did not result in elevated rates of apoptosis in either primary tumors or breast cancer bone metastases, as assessed with immunostaining for cleaved caspase-3 (Additional file 12).

Finally, we did not observe any differences in breast cancer cell apoptosis in response to a stimulator of cGMP production (A-350619 hydrochloride) in control or ABCC5 knockdown cells (data not shown). These observations suggest that ABCC5 does not promote breast cancer proliferation and survival in end-stage bone metastases through a mechanism that involves cGMP efflux and reduced PKG activation.

Elevated cGMP levels have also been shown to modulate the expression of matrix metalloproteinases (MMPs), although this regulation appears complex. Some studies suggest that elevated cGMP levels can induce the expression of MMP-2 and MMP-9 [72,73], whereas others argue that MMP-9 expression or secretion is suppressed by increasing cGMP concentrations [74,75]. Whether breast cancer cells that express ABCC5, and thus maintain low cytoplasmic cGMP levels through active efflux, are associated with elevated MMP-9 expression or secretion requires further investigation.

One interesting result was the observation that the density of TRAP-positive osteoclasts was reduced in lesions formed by breast cancer cells harboring reduced ABCC5 levels compared with bone metastases arising from control cells. Consistent with these observations, conditioned media from ABCC5 knockdown cells were less efficient in inducing *in vitro *osteoclast differentiation compared with media collected from cells harboring scrambled shRNA. It should be noted that the diminishment of osteoclastogenesis was not complete, and, in the case of one ABCC5 shRNA (shRNA 1), only trended toward a decrease in osteoclastogenesis. These results raise the possibility that the substrate of ABCC5 might directly influence osteoclast differentiation; however, it is also conceivable that the cargo that is pumped out of breast cancer cells by ABCC5 indirectly influences osteoclastogenesis through an intermediate cell type present in the bone microenvironment.

Previous studies showed that high levels of cGMP can negatively regulate the ability of osteoclasts to resorb bone, disrupting their attachment to the bone surface and preventing efficient secretion of HCl [76,77]. However, an important phase of the bone resorption mediated by osteoclasts is their ability to break the sealing zone, detach from the bone surface, migrate to a new area of bone, and reinitiate bone resorption. Indeed, nitric oxide or cGMP analogues have been shown to stimulate osteoclast migration [78]. Thus, it is conceivable that locally elevated levels of cGMP, by virtue of a growing breast cancer metastasis, could contribute to enhanced osteoclast migration. The reduction of ABCC5 could diminish cGMP efflux, leading to impaired osteoclast motility and decreased bone resorption. This hypothesis might explain the specific requirement for ABCC5 expression in breast cancer cells that metastasize to the bone. Alternatively, an as-yet-unidentified ABCC5 cargo may be responsible for enhanced osteoclast differentiation and motility that leads to the formation of osteolytic breast cancer metastases in bone.

## Conclusions

We identified *ABCC5 *as a gene that is overexpressed in breast cancer bone metastases compared with primary breast tumors. This protein was also highly expressed in human and mouse cells breast cancer cell lines that are highly metastatic to bone. Finally, *ABCC5 *was functionally validated in *in vivo *models to be an important mediator in breast cancer outgrowth in this organ. *ABCC5 *functions to promote osteolytic bone destruction through the recruitment and enhanced formation of osteoclasts. Hence, *ABCC5 *is a novel candidate mediator of breast cancer bone metastasis, which may be a potential target for the development of treatment for this detrimental disease.

## Abbreviations

ABC transporter: ATP-binding cassette transporter; aRNA: amplified RNA; cAMP: cyclic adenosine monophosphate; cDNA: complementary DNA; cGMP: cyclic guanosine monophosphate; CK: cytokeratin; EDTA: ethylenediaminetetraacetic acid; IHC: immunohistochemistry; IF: immunofluorescence; LCM: laser-capture microdissection; μCT: micro-computed tomography; MIF: macrophage migration inhibitory factor; MMP: matrix metalloproteinase; PKG: protein kinase G; PVDF: polyvinylidene fluoride; RT-qPCR: reverse transcription quantitative polymerase chain reaction; SDS-PAGE: sodium dodecylsulfate polyacrylamide gel electrophoresis; shRNA: short-hairpin RNA; TRAP: tartrate-resistant acid phosphatase.

## Competing interests

The authors declare that they have no competing interests.

## Authors' contributions

AAM carried out the LCM, expression analysis, and experiments focused on ABCC5 function. EA and MC collected the bone-trephine biopsies from breast cancer patients with known bone involvement. ZD performed immunohistochemistry and immunofluorescence staining. KT and SVK conducted the *in vitro *osteoclastogenesis experiments. SC and MH conducted the gene-expression analysis. AO identified regions of primary tumors and bone metastases suitable for LCM and scored the ABCC5 IHC staining. NB and MP generously provided primary breast tumor material. VO assisted in intracardiac injection of mammary tumor cells. GLS generously provided ABCC5 antibody. AAM and PMS designed the experiments, interpreted the results, and prepared the manuscript. All authors read and approved the final manuscript.

## Supplementary Material

Additional file 1 **Supplemental Materials and Methods**. This file contains the experimental procedures not presented in the main text. Click here for file

Additional file 2 **Supplemental Table 1. Clinical data of the breast cancer samples**. This table describes the clinical data of the breast cancer samples used in the study. Click here for file

Additional file 3 **Figure S1: Histologic appearance of primary breast tumors and breast cancer bone metastases**. All of the samples used for the study were reviewed by a breast pathologist. Bone metastases samples primarily consist of the malignant mammary epithelium and stroma. The primary tumor material displays more complexity. All of the primary breast tumor samples contained invasive epithelial cells and stroma. In addition, some of the samples included ductal carcinoma *in situ *(DCIS) as well as normal and hyperplastic mammary ducts. Scale bar represents 50 μm and applies to all the images. Click here for file

Additional file 4 **Figure S2: Human breast cancer metastases express basal keratins or coexpress basal and luminal keratins**. The expression of cytokeratin 8/18 (CK8/18) and cytokeratin 5 (CK5) in breast cancer bone metastases was assessed with immunofluorescence. All of the bone metastases stained positive for the myoepithelial marker CK5, implying their basal-like phenotype. Three of five bone metastases also stained positive for the luminal marker, CK8/18. The images were taken on a confocal microscope under the 63× objective. The scale bar represents 20 μm and applies to all the images. Click here for file

Additional file 5 **Microarray data**. This is a list of differentially expressed probes between bone-trephine biopsies from breast cancer patients and primary breast tumors metastatic to skeleton. Click here for file

Additional file 6 **Figure S3: Categories of genes that are differentially expressed in the breast cancer bone metastases compared with the primary breast tumors that were metastatic to bone**. Application of filter criteria described in Materials and Methods resulted in a list of 244 overexpressed and 185 underexpressed probes in breast cancer skeletal metastases compared with primary breast tumors. This list was further condensed to 118 upregulated and 82 downregulated genes when only unique genes with described functions were considered. These genes were subcategorized on the basis of their known functions. Click here for file

Additional file 7 **Figure S4: Agilent gene-expression data of genes selected for RT-qPCR validation**. A subset of candidate genes that were differentially expressed between breast cancer bone metastases and primary tumors is shown. Several members of the ATP-binding cassette (ABC) transporter family were found to be overexpressed in breast cancer bone metastases relative to the primary tumors metastatic to bone. Click here for file

Additional file 8 **Figure S5: RT-qPCR validation of genes that are differentially expressed between primary breast tumors and breast cancer bone metastases**. The Agilent gene-expression data were validated at the message level with RT-qPCR. A high degree of concordance between the Agilent microarray expression data (Additional File 7, Figure S4) and the RT-qPCR analysis was observed. Click here for file

Additional file 9 **Figure S6: Reduced ABCC5 expression does not alter growth characteristics of MDA-MB-231 human or 4T1 mouse breast cancer cells**. **(A) ***In vitro *growth curves for 1833-BM1 cells expressing either scrambled (Scr shRNA) or *ABCC5 *specific short-hairpin RNAs (*ABCC5 *shRNA) are shown. The average of three independent wells is presented for each time point. The error bars denote the standard error of the mean (SEM). **(B) **Tumor growth curves after mammary fat pad injection are shown. Mammary tumor growth was measured biweekly for 29 days in mice injected with 1833-BM1 expressing Scr shRNA (*n *= 14) or *ABCC5 *shRNAs (*n *= 16). Error bars signify the SEM for each time point. **(C) ***In vitro *growth curves are shown for 4T1-derived breast cancer cells expressing either scrambled (Scr shRNA) or *ABCC5 *specific short-hairpin RNAs (*ABCC5 *shRNA). The average of three independent wells is presented for each time point. The error bars denote the SEM. **(D) **Growth of 4T1-derived mammary tumors in syngeneic mice after mammary fat pad injection is shown. Mammary tumor growth was measured biweekly for 36 days in mice injected with 4T1 cells expressing Scr shRNAs (*n *= 10) or two independent *ABCC5 *shRNAs [shRNA 1 (*n *= 10) or shRNA 2 (*n *= 10)]. Error bars signify the SEM for each time point. Click here for file

Additional file 10 **Figure S7: Reduced ABCC5 expression does not alter spontaneous lung metastasis burden in mice bearing 4T1-derived mammary tumors**. Lungs from mice bearing 4T1 tumors expressing Scr shRNA (*n *= 5) or two independent *ABCC5 *shRNAs (shRNA 1 (*n *= 5) or shRNA 2 (*n *= 5)) extracted at end point and stained with H&E. The number of lesions per lung **(A) **and the total lesion area per lung **(B) **were analyzed. The error bars represent the standard error of the mean and apply to all the graphs. Click here for file

Additional file 11 **Figure S8: Reduced ABCC5 expression does not alter the proliferative index of human MDA-MB-231 or mouse 4T1 primary tumors nor bone metastases at end stage**. **(A) **Primary tumors derived from mice injected with 1833-BM1 cells expressing Scr shRNA (*n *= 5) or *ABCC5 *shRNA (*n *= 5) on day 29 after injection were stained against Ki67. **(B) **Primary tumors derived from mice injected with 4T1 cells expressing Scr shRNAs (*n *= 5) or two independent *ABCC5 *shRNAs (shRNA1 (*n *= 5) or shRNA2 (*n *= 5)) on day 36 after injection were stained against Ki67. **(C) **Hindlimbs with bone metastases formed from the intracardiac injection of 1833-BM1 cells expressing Scr shRNA (*n *= 5) or *ABCC5 *shRNA (*n *= 5) on day 21 after inoculation were stained against Ki67. **(D) **Hindlimbs with bone metastases formed from the intracardiac injection of 4T1 cells expressing Scr shRNAs (*n *= 5) or two independent *ABCC5 *shRNAs (shRNA 1 (*n *= 5) or shRNA 2 (*n *= 5)) on day 13 after inoculation were stained against Ki67. Proliferation is expressed as the percentage of Ki67-positive nuclei. The error bars represent the standard error of the mean and apply to all the graphs. Click here for file

Additional file 12 **Figure S9: Reduced ABCC5 expression does not alter the apoptosis of human MDA-MB-231 or mouse 4T1 primary tumors or bone metastases at end stage**. **(A) **Primary tumors derived from mice injected with 1833-BM1 cells expressing Scr shRNA (*n *= 5) or *ABCC5 *shRNA (*n *= 5) on day 29 after injection were stained against cleaved caspase-3. **(B) **Primary tumors derived from mice injected with 4T1 cells expressing Scr shRNAs (*n *= 5) or two independent *ABCC5 *shRNAs (shRNA 1 (*n *= 5) or shRNA 2 (*n *= 5)) on day 36 after injection were stained against cleaved caspase-3. **(C) **Hindlimbs with bone metastases formed from the intracardiac injection of 1833-BM1 cells expressing Scr shRNA (*n *= 5) or *ABCC5 *shRNA (*n *= 5) on day 21 after inoculation were stained against cleaved caspase-3. **(D) **Hindlimbs with bone metastases formed from the intracardiac injection of 4T1 cells expressing Scr shRNAs (*n *= 5) or two independent *ABCC5 *shRNAs (shRNA1 (*n *= 5) or shRNA2 (*n *= 5)) on day 13 after inoculation were stained against cleaved caspase-3. Apoptosis is expressed as the number of cleaved caspase-3-positive nuclei per 1,000 cells. The error bars represent the standard error of the mean and apply to all the graphs. Click here for file
