# Supplementary material for: ABCC5 supports osteoclast formation and promotes breast cancer metastasis to bone
Source: Breast Cancer Res. 2012 Nov 22;14(6):R149. doi: 10.1186/bcr3361 (PMC4053136; doi:10.1186/bcr3361)
Supplement: Additional file 1 — Supplemental Materials and Methods. This file contains the experimental procedures not presented in the main text. [file bcr3361-S1.DOCX]

**Supplemental Materials and Methods**

**Immunofluorescence staining**

OCT-embedded primary breast tumours and bone trephine biopsies were cut at 10μm thickness and fixed in 2% paraformaldehyde. The sections were blocked with 2% BSA and 5% NGS and incubated overnight at 4^o^C with the following primary antibodies: Cytokeratin 8/18 (1:250, Fitzgerald, 20R-CP004) and Cytokeratin 5 (1:1000, Covance, PRB-160P). The sections were then incubated with Cy2 or Cy3 conjugated secondary antibodies respectfully. Standard DAPI counterstain was performed to identify cellular nuclei.

**Ki67, cleaved-caspase and CD31 immunohistochemistry**

Primary mammary tumours and hindlimbs were excised from mice and fixed overnight in 4% paraformaldehyde. Bones were then decalcified in a solution of 14.5% ethylenediaminetetraacetic acid (EDTA) and 15% glycerol for 4 weeks. Tissues were then paraffin embedded and sectioned. Immunohistochemistry was performed on 5μm sections with the following antibodies: Ki67 (1:1000 dilution; Cat #: ab15581, Abcam), cleaved caspase-3 (1:200 dilution; Cat. #: 9661 Cell Signaling). The appropriate Biotin-SP-conjugated anti-IgG secondary antibodies were purchased from Jackson Laboratories (Bar Harbor, ME). Sections were developed with 3-3-diaminobenzidine-tetrahydrochloride and counterstained with hematoxylin. Slides were scanned using a Scanscope XT digital slide scanner (Aperio, Vista, CA, USA) and analyzed with Imagescope software (Aperio) using the IHC nuclear algorithm.

**Real-Time Quantitative Reverse Transcription PCR**

Validation of candidate genes by RT-qPCR was performed as described in the Material and Methods section of the manuscript. The sequences of primers used for each validated target are as follows:

| Target Gene | Gene ID | Primer Sequence | Primer Orientation  (F: Forward; R: Reverse) |
| --- | --- | --- | --- |
| *ABCA5* | NM_018672 | 5’ atgctgtggttgtgtgtttt 3’ | F |
|  |  | 5’ gaagcactgtttctgaatgg 3’ | R |
| *ABCC5* | NM_005688 | 5’ tcatcaattgaatggtcagc 3’ | F |
|  |  | 5’ cattttcagatttttgcttcc 3’ | R |
| *ABCG2* | NM_004827 | 5’ ctcacaattgcctacctgaa 3’ | F |
|  |  | 5’ cttcaatcaaagtgcttctttt 3’ | R |
| *ATP1B2* | NM_001678 | 5’ ttctctggcttcttttagca 3’ | F |
|  |  | 5’ agagatggagccaagacatt 3’ | R |
| *BCL2* | NM_000633 | 5’ tctgcaacactgtacacataaaa 3’ | F |
|  |  | 5’ gcaatgtgactttttccaac 3’ | R |
| *CMTM6* | NM_017801 | 5’ agaaatggagtcatgtgctg 3’ | F |
|  |  | 5’ aggactctcaagatcctgct 3’ | R |
| *EPAS1* | NM_001430 | 5’ tgctggtatttgatttcctg 3’ | F |
|  |  | 5’ tcgttacgttgacaggtagg 3’ | R |
| *MARCKS* | NM_002356 | 5’ ggagaagctttgaccaattt 3’ | F |
|  |  | 5’ ttttcgcttttagtgtgctc 3’ | R |
| *NEDD4L* | NM_015277 | 5’ tggaaggttgtggttgtaaa 3’ | F |
|  |  | 5’ aaaaggaaccaacactcagc 3’ | R |
| *PTPRM* | NM_002845 | 5’ cagatttgtattgtttccaagg 3’ | F |
|  |  | 5’ aaacacatggaaaagctctg 3’ | R |
| *RUNX2* | NM_001015051 | 5’ aacaaccacagaaccacaag 3’ | F |
|  |  | 5’ tgcagccttaaatgactctg 3’ | R |
| *SLC9A9* | NM_173653 | 5’ ggtatcaggcacactgctat 3’ | F |
|  |  | 5’ atgctccaccacttgtcata 3’ | R |
| *SLIT3* | NM_003062 | 5’ ttttaccaagttttgtgttgtg 3’ | F |
|  |  | 5’ cttcctcagacaggctatca 3’ | R |
| *TGFBR2* | NM_001024847 | 5’ caaaagtctcaagcacttatttt 3’ | F |
|  |  | 5’ actaggaatgggaacaggag 3’ | R |
| *BID* | NM_197966 | 5’ aagacaggctggaagatagc 3’ | F |
|  |  | 5’ ggccattcaaatacgtgtaa 3’ | R |
| *BSPRY* | NM_017688 | 5’ caaatgatgtgtgtggtgtt 3’ | F |
|  |  | 5’ aaaagtttggacacctgtga 3’ | R |
| *CD44* | NM_000610 | 5’ gacatgaagattggggtgta 3’ | F |
|  |  | 5’ attcgcaatgaaacaatcag 3’ | R |
| *ECE2* | NM_032331 | 5’ gctcctcaggtagttggaat 3’ | F |
|  |  | 5’ tctggatctagttggagctg 3’ | R |
| *HTRA3* | NM_053044 | 5’ cagggaacgagtgtgtctc 3’ | F |
|  |  | 5’ gaaaaccgtgtgcttgtaaa 3’ | R |
| *MIF* | NM_002415 | 5’ ctggaacaactccaccttc 3’ | F |
|  |  | 5’ gtttatttctccccaccaga 3’ | R |
| *PHPT1* | NM_014172 | 5’ ctttgcctgcactcctct 3’ | F |
|  |  | 5’ gtggcaacacgtttaattct 3’ | R |
| *TESK1* | NM_006285 | 5’ ggacagagcacttccagtc 3’ | F |
|  |  | 5’ ccaggcagttttattgaaatc 3’ | R |
| *TMSB10* | NM_021103 | 5’ cgctcttttgtttcttgct 3’ | F |
|  |  | 5’ ggtttgtctgccattttctt 3’ | R |

***In vitro* breast cancer cell growth curves**

Human 1833-BM1 and mouse 4T1 cells (10^4^ cells) were seeded in 24-well culture dishes, allowed to attach and were stained with a solution of crystal violet (0.5% w/v), sodium chloride (0.8% w/v), formal saline (0.5% v/v) and absolute ethanol (49% v/v). Cells were then lysed with 33% acetic acid and the absorbency of the released dye was measured using a spectrophotometer at a wavelength of 570nm. Cells were stained daily for 5 (mouse cell lines) and 6 (human cell lines) days.

***In vivo* mammary tumour growth and spontaneous lung metastasis**

Age-matched (8 weeks) SCID/beige and BALB/c female mice were purchased from Charles River Laboratories. The mice were housed in facilities managed by the McGill University Animal Resources Centre, and all animal experiments were conducted under the McGill University–approved Animal Use Protocol in accordance with guidelines established by the Canadian Council on Animal Care. For primary tumour growth assays, 1833-BM1 (10^6^ cells) and 4T1 (10^4^ cells) breast cancer cell populations were first suspended in a 50:50 solution of 1 x PBS and growth-factor-reduced matrigel (Cat. #: 354234, BD Biosciences) and subsequently injected into the number 4 abdominal mammary fat pad, in a total volume of 50μl [[1](#_ENREF_1)]. Tumour volumes were monitored by biweekly calliper measurements and calculated using the following formula: πLW^2^/6, where L is the length and W is the width of the tumour. Animals were sacrificed 29 (human) and 36 (mouse) days post injection.

To quantify spontaneous lung metastasis, all lobes of the lung were extracted from mice bearing 4T1-derivative mammary tumours, perfused with a mixture of 50:50 4% paraformaldehyde and O.C.T. and fixed overnight in 4% paraformaldehyde. The lungs were embedded in paraffin 4 step sections were taken, with 100 µm between each step. The slides were scanned using Aperio Slide Scanner. The metastases were then individually outlined and the area of metastases was calculated using bundled software. The number of lesions and area/lesion were scored for each step section and an average was calculated over the 4 step sections.

**Supplementary References**

1. Mourskaia AA, Dong Z, Ng S, Banville M, Zwaagstra JC, O'Connor-McCourt MD, Siegel PM: **Transforming growth factor-beta1 is the predominant isoform required for breast cancer cell outgrowth in bone**. *Oncogene* 2009, **28**(7):1005-1015.
