# Supplementary material for: ABCC5 supports osteoclast formation and promotes breast cancer metastasis to bone
Source: Breast Cancer Res. 2012 Nov 22;14(6):R149. doi: 10.1186/bcr3361 (PMC4053136; doi:10.1186/bcr3361)
Supplement: Additional file 2 — Supplemental Table 1. Clinical data of the breast cancer samples. This table describes the clinical data of the breast cancer samples used in the study. [file bcr3361-S2.PDF]

Supplemental Table 1: Clinical data of the breast cancer samples

Patient ID	Primary tumor ER/ PR/HER2 status	Primary tumor histopathology	Relapse location	
BT-086	neg./neg./neg.	Invasive ductal carcinoma	**Bone**, pleura	Primary tumors
BT-124	pos./pos./neg.	Invasive ductal carcinoma	Lymph node, **bone**, liver	
BT-127	pos./pos./neg.	Invasive ductal carcinoma	**Bone**, meninges	
BT-136	pos./pos./neg.	Invasive ductal carcinoma	**Bone**	
BT-238	pos./pos./neg.	Invasive ductal carcinoma	**Bone**	
BM-001	95%/<5%/neg.	Invasive ductal carcinoma	Lymph node, **bone**	Bone metastases
BB-005	95%/95%/neg.	Invasive ductal carcinoma	**Bone**	
BM-006	pos./pos./unknown	Invasive ductal carcinoma	Local, **bone**	
BM-007	pos./pos./neg.	Invasive ductal carcinoma	Liver, **bone**	
BB-018	neg./neg./neg.	Invasive ductal carcinoma	**Bone**, other n/a	
