# Supplementary figures and images for: ABCC5 supports osteoclast formation and promotes breast cancer metastasis to bone
Source: Breast Cancer Res. 2012 Nov 22;14(6):R149. doi: 10.1186/bcr3361 (PMC4053136; doi:10.1186/bcr3361)

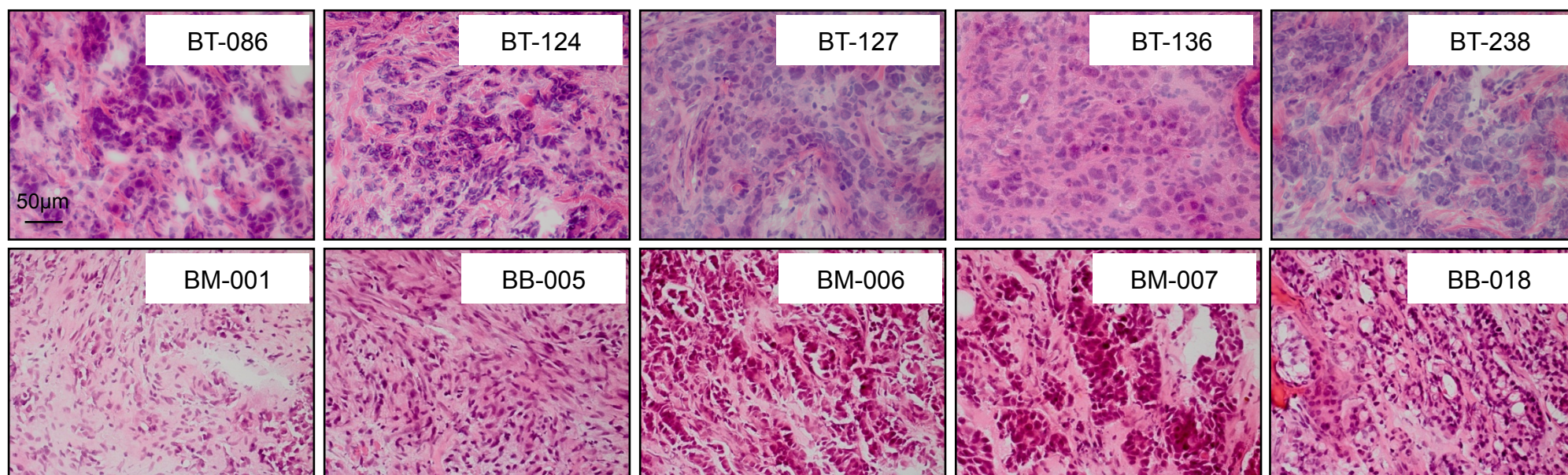

Supplement: Additional file 3 — Figure S1: Histologic appearance of primary breast tumors and breast cancer bone metastases. All of the samples used for the study were reviewed by a breast pathologist. Bone metastases samples primarily consist of the malignant mammary epithelium and stroma. The primary tumor material displays more complexity. All of the primary breast tumor samples contained invasive epithelial cells and stroma. In addition, some of the samples included ductal carcinoma in situ (DCIS) as well as normal and hyperplastic mammary ducts. Scale bar represents 50 μm and applies to all the images. [file bcr3361-S3.PDF]

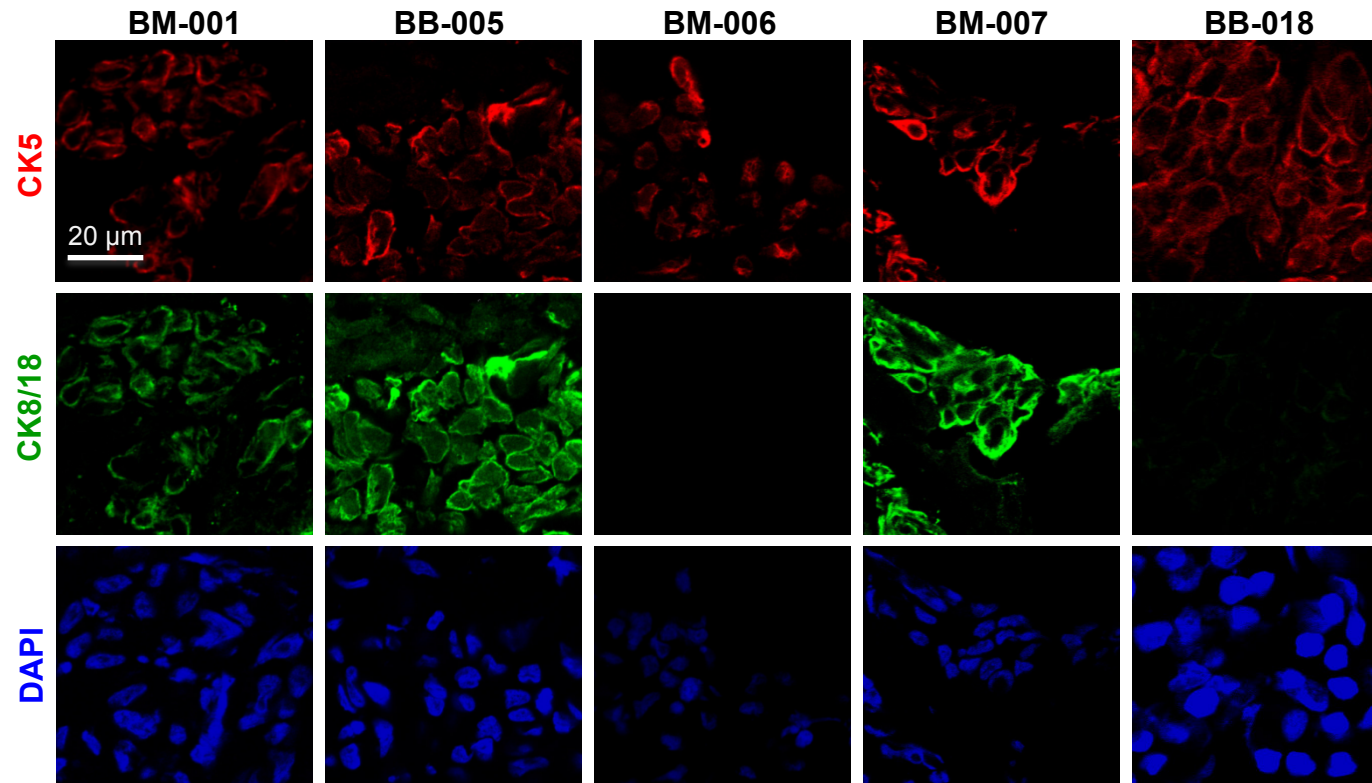

Mourskaia *et al.*, Supplementary Figure 2

Supplement: Additional file 4 — Figure S2: Human breast cancer metastases express basal keratins or coexpress basal and luminal keratins. The expression of cytokeratin 8/18 (CK8/18) and cytokeratin 5 (CK5) in breast cancer bone metastases was assessed with immunofluorescence. All of the bone metastases stained positive for the myoepithelial marker CK5, implying their basal-like phenotype. Three of five bone metastases also stained positive for the luminal marker, CK8/18. The images were taken on a confocal microscope under the 63× objective. The scale bar represents 20 μm and applies to all the images. [file bcr3361-S4.PDF]

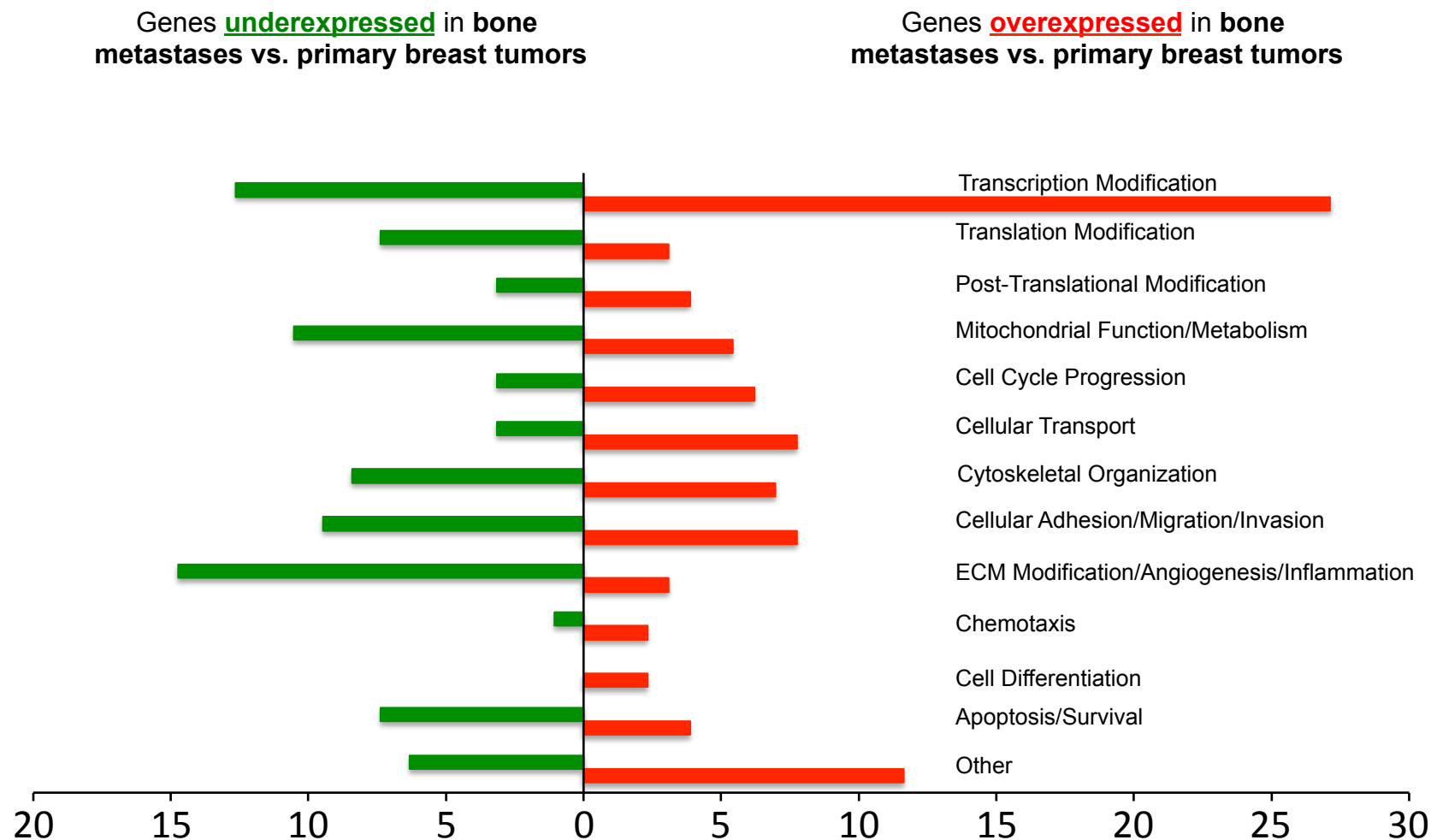

Supplement: Additional file 6 — Figure S3: Categories of genes that are differentially expressed in the breast cancer bone metastases compared with the primary breast tumors that were metastatic to bone. Application of filter criteria described in Materials and Methods resulted in a list of 244 overexpressed and 185 underexpressed probes in breast cancer skeletal metastases compared with primary breast tumors. This list was further condensed to 118 upregulated and 82 downregulated genes when only unique genes with described functions were considered. These genes were subcategorized on the basis of their known functions. [file bcr3361-S6.PDF]

# Microarray Gene Expression Data

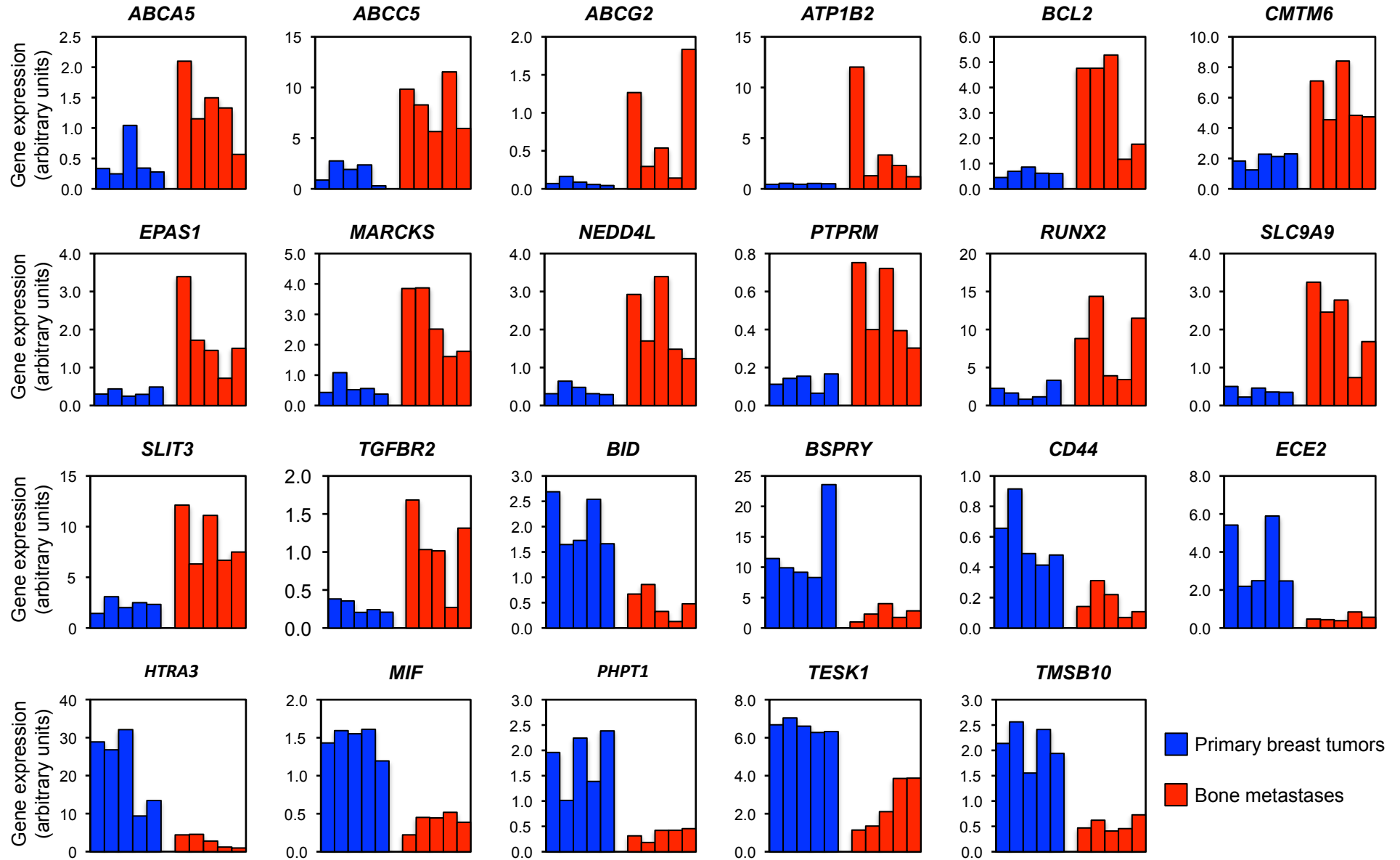

Supplement: Additional file 7 — Figure S4: Agilent gene-expression data of genes selected for RT-qPCR validation. A subset of candidate genes that were differentially expressed between breast cancer bone metastases and primary tumors is shown. Several members of the ATP-binding cassette (ABC) transporter family were found to be overexpressed in breast cancer bone metastases relative to the primary tumors metastatic to bone. [file bcr3361-S7.PDF]

## RT-qPCR Expression Data

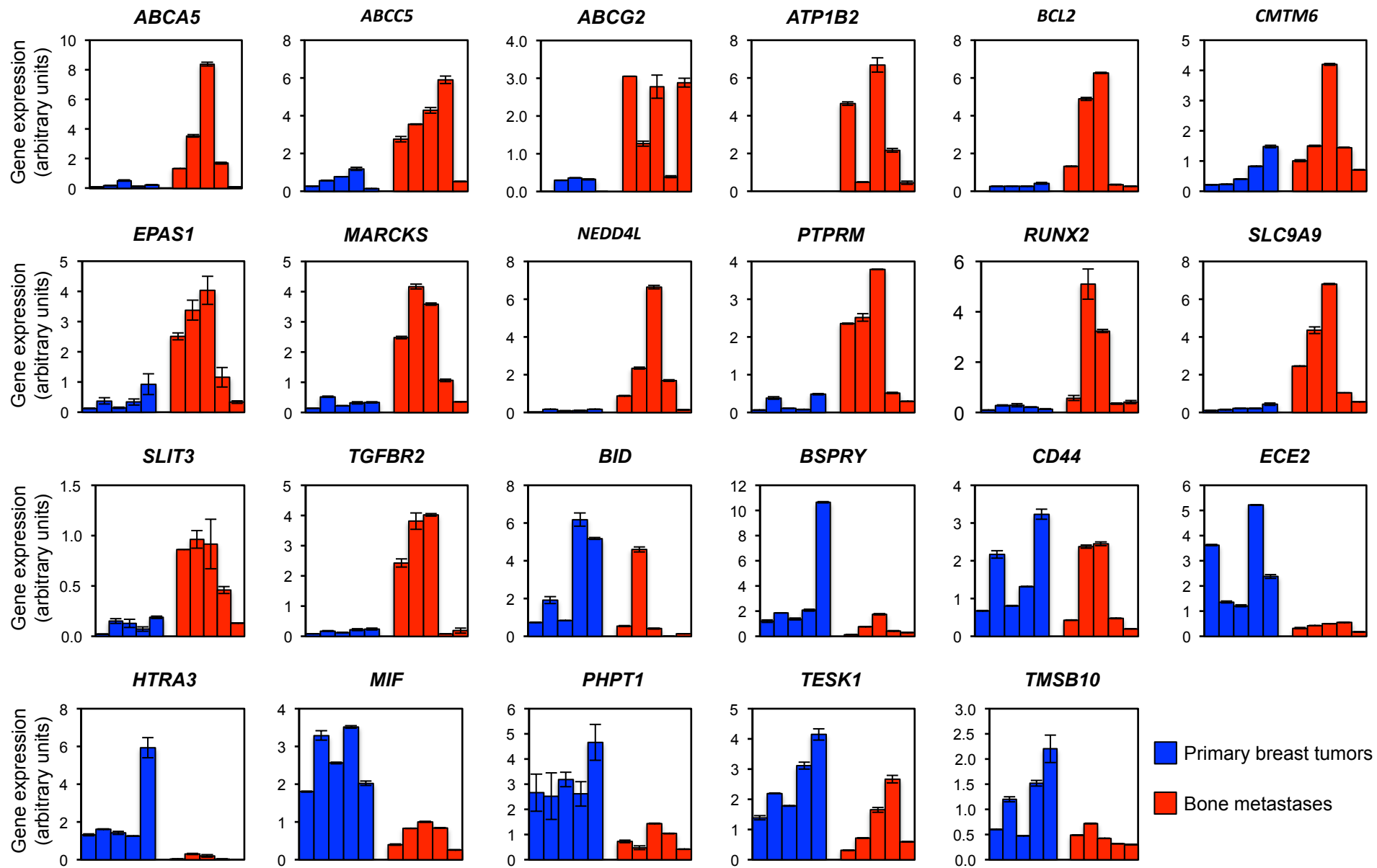

Supplement: Additional file 8 — Figure S5: RT-qPCR validation of genes that are differentially expressed between primary breast tumors and breast cancer bone metastases. The Agilent gene-expression data were validated at the message level with RT-qPCR. A high degree of concordance between the Agilent microarray expression data (Additional File 7, Figure S4) and the RT-qPCR analysis was observed. [file bcr3361-S8.PDF]

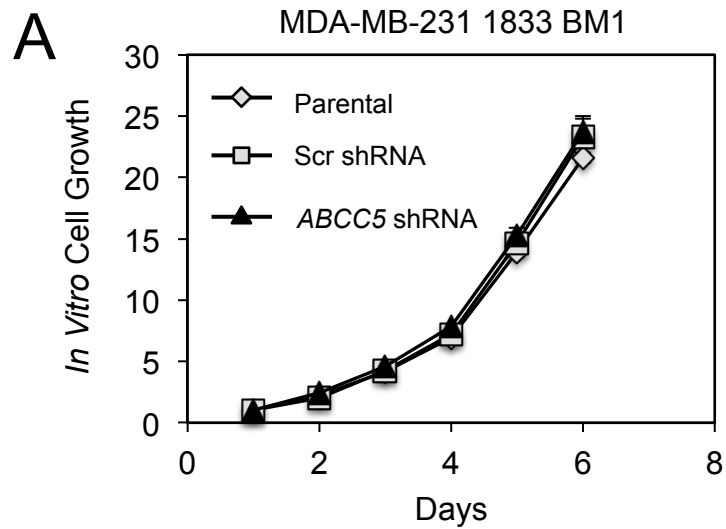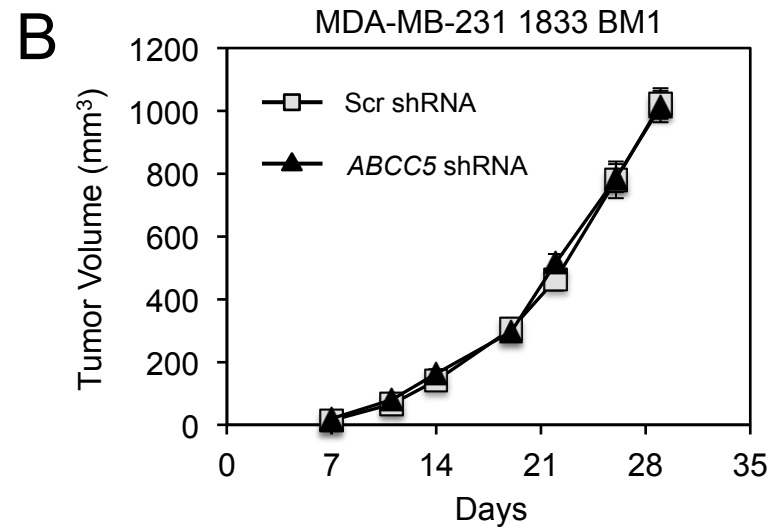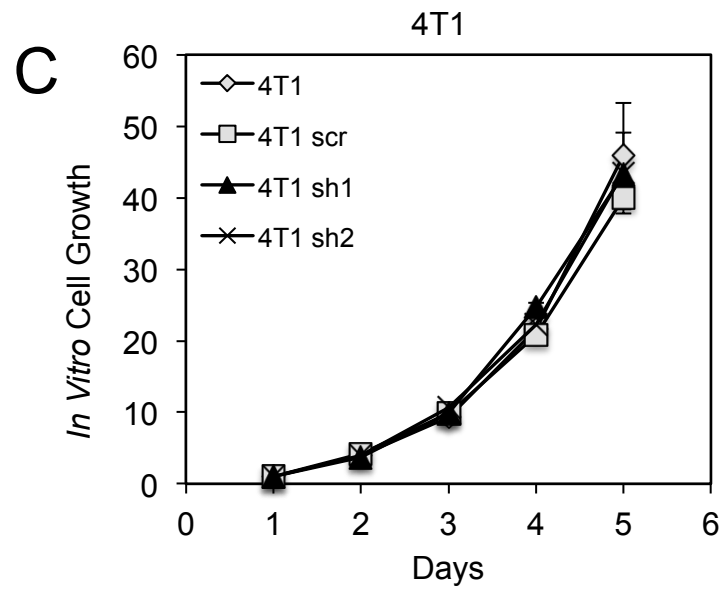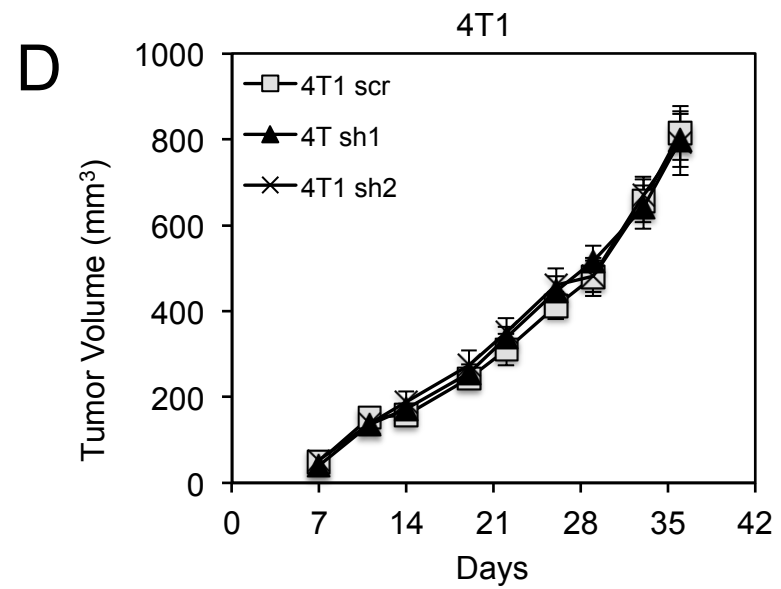

Supplement: Additional file 9 — Figure S6: Reduced ABCC5 expression does not alter growth characteristics of MDA-MB-231 human or 4T1 mouse breast cancer cells. (A) In vitro growth curves for 1833-BM1 cells expressing either scrambled (Scr shRNA) or ABCC5 specific short-hairpin RNAs (ABCC5 shRNA) are shown. The average of three independent wells is presented for each time point. The error bars denote the standard error of the mean (SEM). (B) Tumor growth curves after mammary fat pad injection are shown. Mammary tumor growth was measured biweekly for 29 days in mice injected with 1833-BM1 expressing Scr shRNA (n = 14) or ABCC5 shRNAs (n = 16). Error bars signify the SEM for each time point. (C) In vitro growth curves are shown for 4T1-derived breast cancer cells expressing either scrambled (Scr shRNA) or ABCC5 specific short-hairpin RNAs (ABCC5 shRNA). The average of three independent wells is presented for each time point. The error bars denote the SEM. (D) Growth of 4T1-derived mammary tumors in syngeneic mice after mammary fat pad injection is shown. Mammary tumor growth was measured biweekly for 36 days in mice injected with 4T1 cells expressing Scr shRNAs (n = 10) or two independent ABCC5 shRNAs [shRNA 1 (n = 10) or shRNA 2 (n = 10)]. Error bars signify the SEM for each time point. [file bcr3361-S9.PDF]

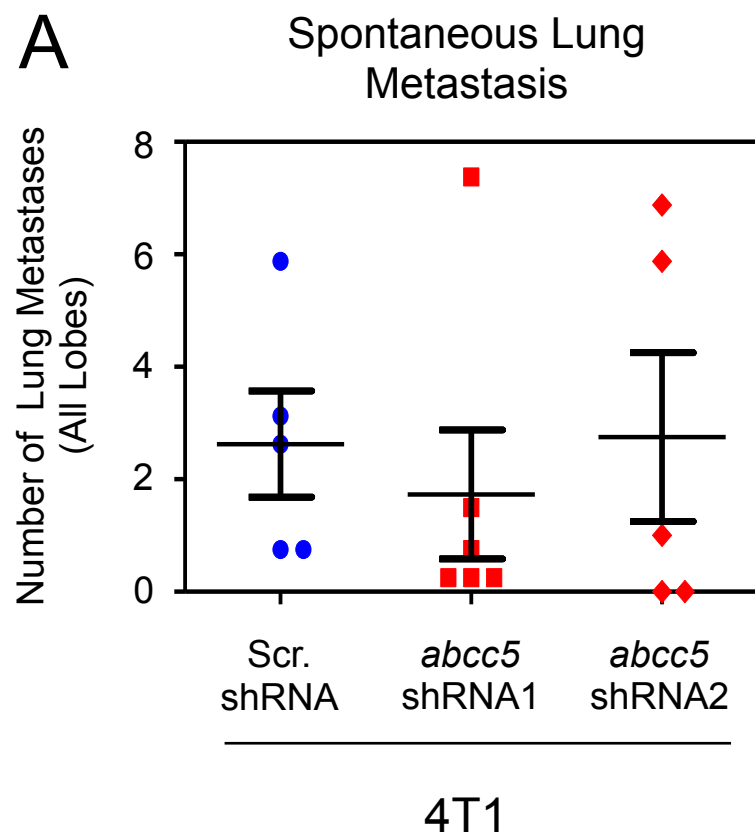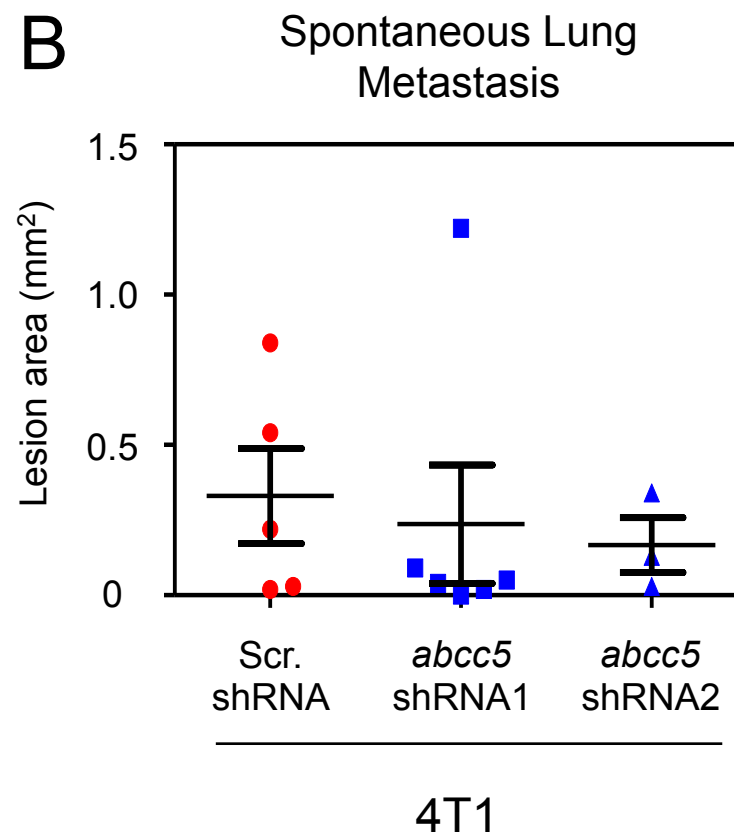

Supplement: Additional file 10 — Figure S7: Reduced ABCC5 expression does not alter spontaneous lung metastasis burden in mice bearing 4T1-derived mammary tumors. Lungs from mice bearing 4T1 tumors expressing Scr shRNA (n = 5) or two independent ABCC5 shRNAs (shRNA 1 (n = 5) or shRNA 2 (n = 5)) extracted at end point and stained with H&E. The number of lesions per lung (A) and the total lesion area per lung (B) were analyzed. The error bars represent the standard error of the mean and apply to all the graphs. [file bcr3361-S10.PDF]

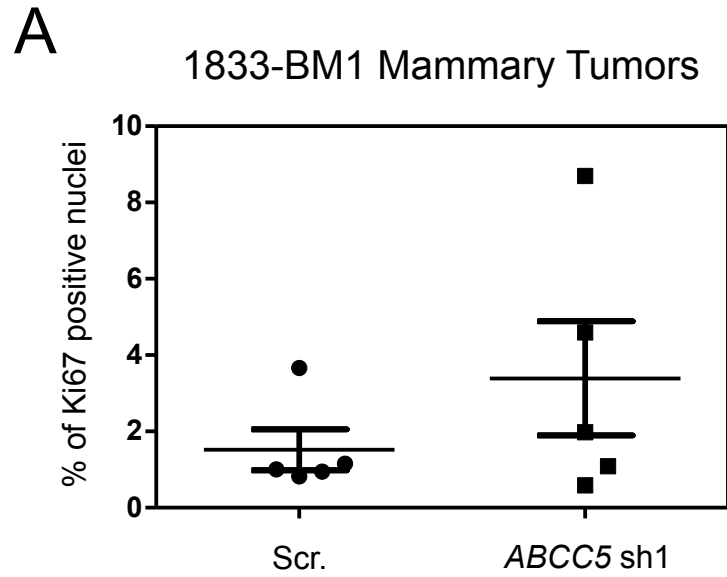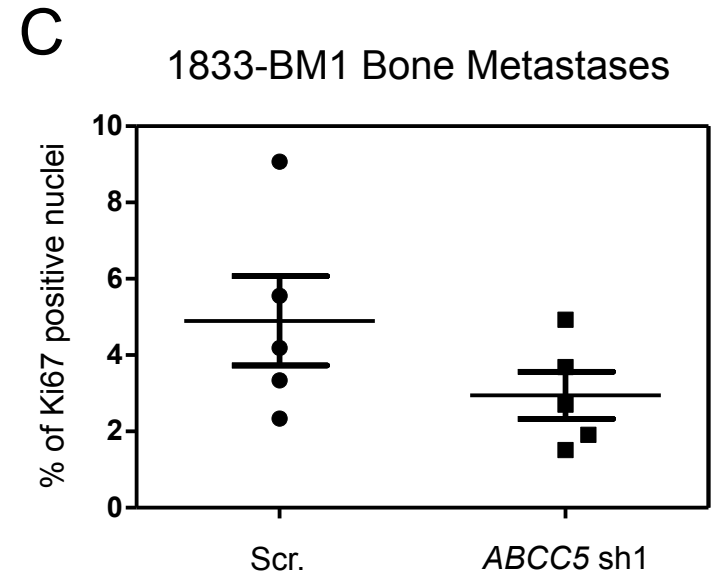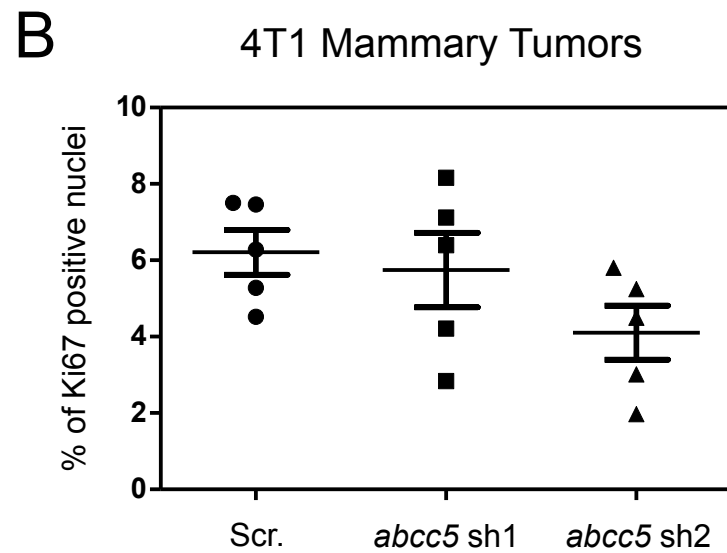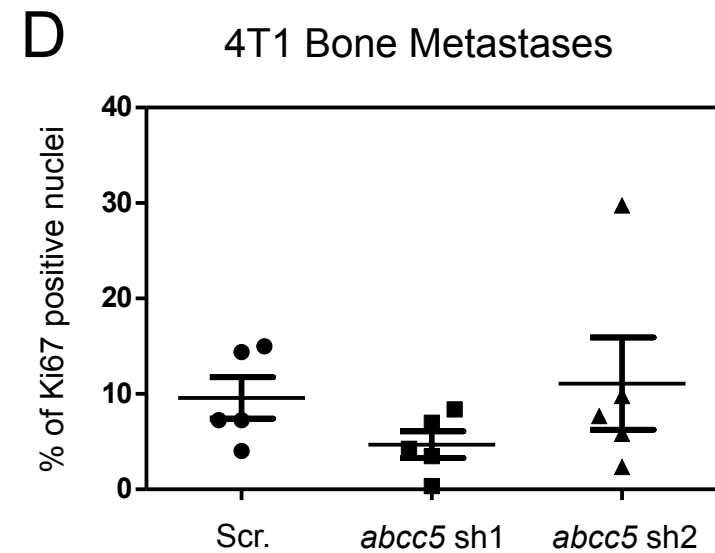

Supplement: Additional file 11 — Figure S8: Reduced ABCC5 expression does not alter the proliferative index of human MDA-MB-231 or mouse 4T1 primary tumors nor bone metastases at end stage. (A) Primary tumors derived from mice injected with 1833-BM1 cells expressing Scr shRNA (n = 5) or ABCC5 shRNA (n = 5) on day 29 after injection were stained against Ki67. (B) Primary tumors derived from mice injected with 4T1 cells expressing Scr shRNAs (n = 5) or two independent ABCC5 shRNAs (shRNA1 (n = 5) or shRNA2 (n = 5)) on day 36 after injection were stained against Ki67. (C) Hindlimbs with bone metastases formed from the intracardiac injection of 1833-BM1 cells expressing Scr shRNA (n = 5) or ABCC5 shRNA (n = 5) on day 21 after inoculation were stained against Ki67. (D) Hindlimbs with bone metastases formed from the intracardiac injection of 4T1 cells expressing Scr shRNAs (n = 5) or two independent ABCC5 shRNAs (shRNA 1 (n = 5) or shRNA 2 (n = 5)) on day 13 after inoculation were stained against Ki67. Proliferation is expressed as the percentage of Ki67-positive nuclei. The error bars represent the standard error of the mean and apply to all the graphs. [file bcr3361-S11.PDF]

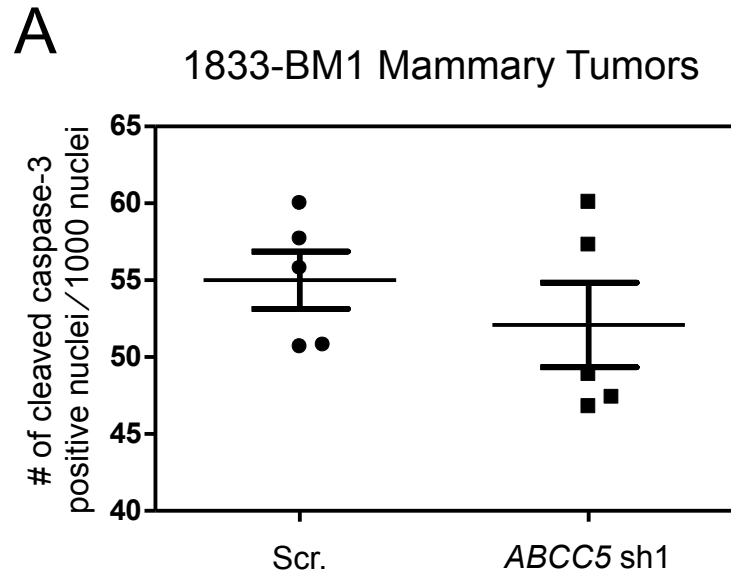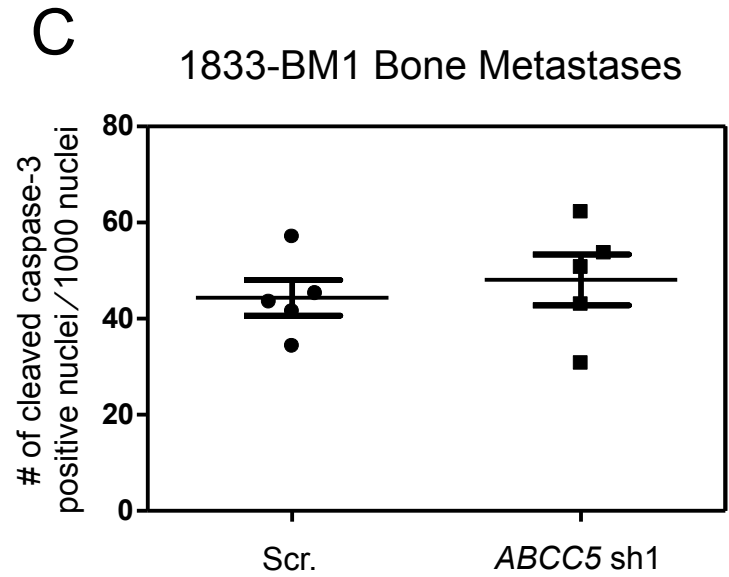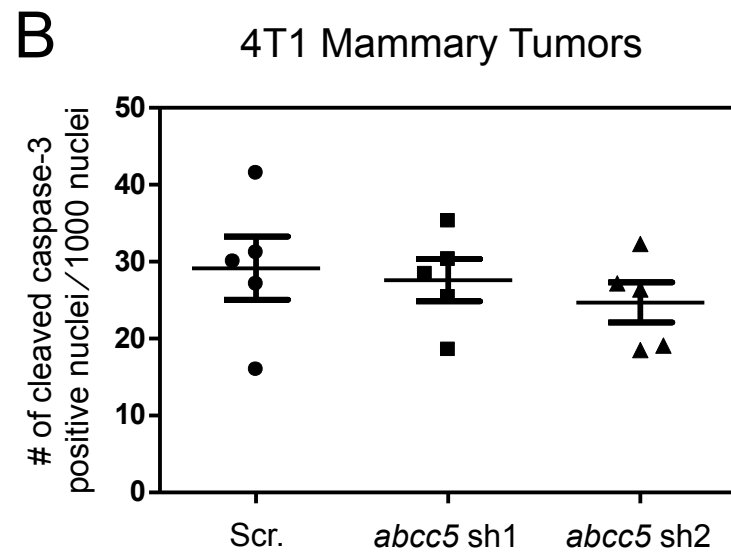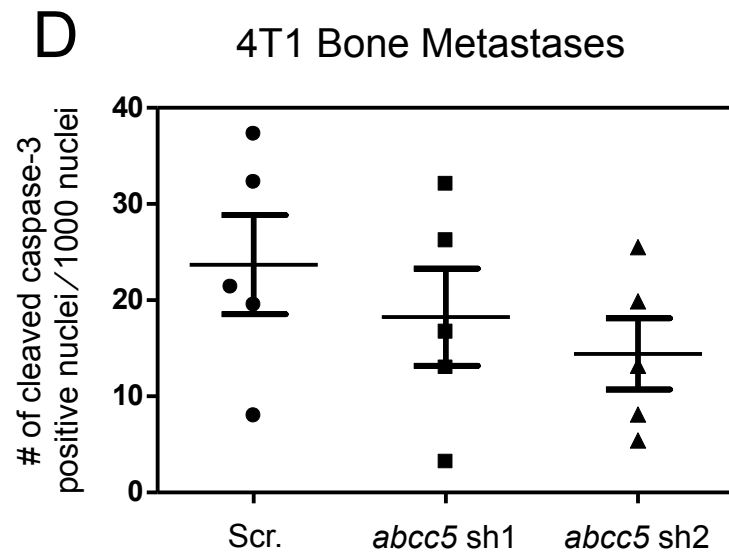

Supplement: Additional file 12 — Figure S9: Reduced ABCC5 expression does not alter the apoptosis of human MDA-MB-231 or mouse 4T1 primary tumors or bone metastases at end stage. (A) Primary tumors derived from mice injected with 1833-BM1 cells expressing Scr shRNA (n = 5) or ABCC5 shRNA (n = 5) on day 29 after injection were stained against cleaved caspase-3. (B) Primary tumors derived from mice injected with 4T1 cells expressing Scr shRNAs (n = 5) or two independent ABCC5 shRNAs (shRNA 1 (n = 5) or shRNA 2 (n = 5)) on day 36 after injection were stained against cleaved caspase-3. (C) Hindlimbs with bone metastases formed from the intracardiac injection of 1833-BM1 cells expressing Scr shRNA (n = 5) or ABCC5 shRNA (n = 5) on day 21 after inoculation were stained against cleaved caspase-3. (D) Hindlimbs with bone metastases formed from the intracardiac injection of 4T1 cells expressing Scr shRNAs (n = 5) or two independent ABCC5 shRNAs (shRNA1 (n = 5) or shRNA2 (n = 5)) on day 13 after inoculation were stained against cleaved caspase-3. Apoptosis is expressed as the number of cleaved caspase-3-positive nuclei per 1,000 cells. The error bars represent the standard error of the mean and apply to all the graphs. [file bcr3361-S12.PDF]
